# Supplementary material for: Therapeutic development of group B Streptococcus meningitis by targeting a host cell signaling network involving EGFR
Source: EMBO Mol Med. 2021 Jan 21;13(3):e12651. doi: 10.15252/emmm.202012651 (PMC7933950; doi:10.15252/emmm.202012651)
Supplement: Supplementary file 1 — Expanded View Figures PDF [file EMMM-13-e12651-s001.pdf]

Expanded View Figures

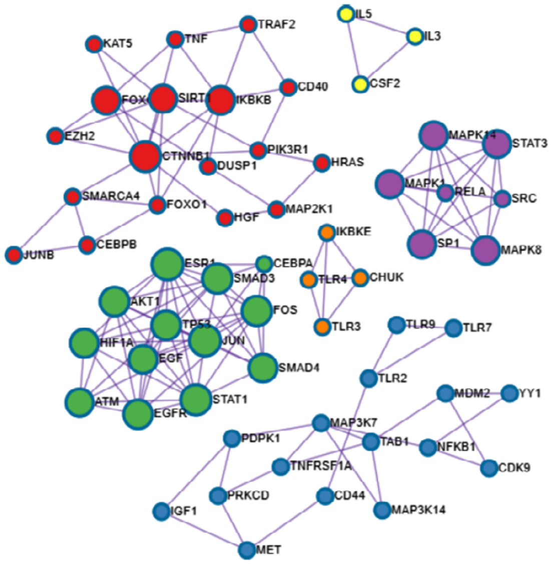

Figure EV1. Protein–protein interaction enrichment analysis of differentially expressed host factors in HBMEC with and without GBS infection.

The protein–protein interaction enrichment analysis results were generated by Metascape.

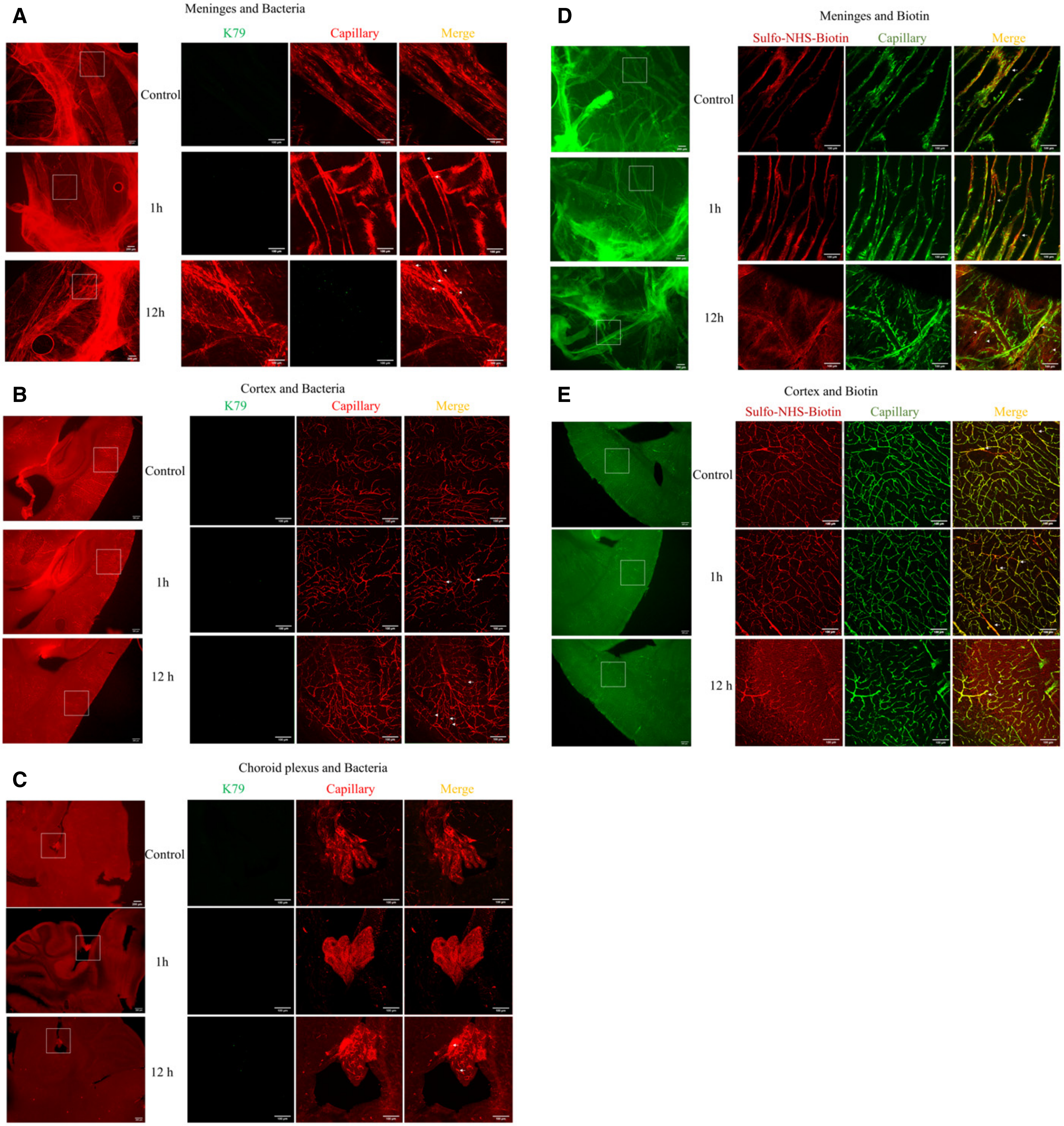

Figure EV2.

 © 2021 The Authors

◀ **Figure EV2. GBS penetration into the meningeal and cortex capillaries and assessment of the blood–brain barrier permeability.**

A–C Wild-type mice received strain GFP-K79 via the tail vein. 1 h and 12 h later, animals were perfused, and the meninges (dura and arachnoid mater), cortex and choroid plexus obtained for demonstration of intravenously injected bacteria. The arrows represent bacteria co-localized with capillaries (e.g., within capillaries), and arrowheads represent bacteria outside the capillaries (e.g., exited from the capillaries). At 1 h following intravenous inoculation, most bacteria were co-localized with the meningeal and cortex capillaries. A few bacteria were found outside the meningeal and cortex capillaries, indicating successful penetration into the brain at this time. Of note, no bacteria were demonstrated in the choroid plexus. At 12 h following intravenous injection, more bacteria outside the meningeal and cortex capillaries were seen, and the presence of bacteria was demonstrated in the choroid plexus. Scale bar = 100 μm. The figures of 1 h infection group were also shown in Fig 1B with enlargement.

D, E Images showing the sulfo-NHS-biotin tracer extravasation. Sulfo-NHS-biotin was administered via intraperitoneal injection 10 min before intracardiac perfusion for assessing the blood–brain barrier permeability, i.e., leakage from the cerebral capillaries. 1 h after intravenous infection with GBS, Sulfo-NHS-biotin was confined to the capillaries of the meninges and cortex (as shown with arrows), which was identical to that in the uninfected control group. 12 h after infection, extravascular Sulfo-NHS-biotin was demonstrated outside the meninges and cortex capillaries, e.g., extravasation (arrowhead), indicative of disruption of the blood–brain barrier permeability. Scale bar = 100 μm. The figures of 1h infection group were also shown in Fig 1C.

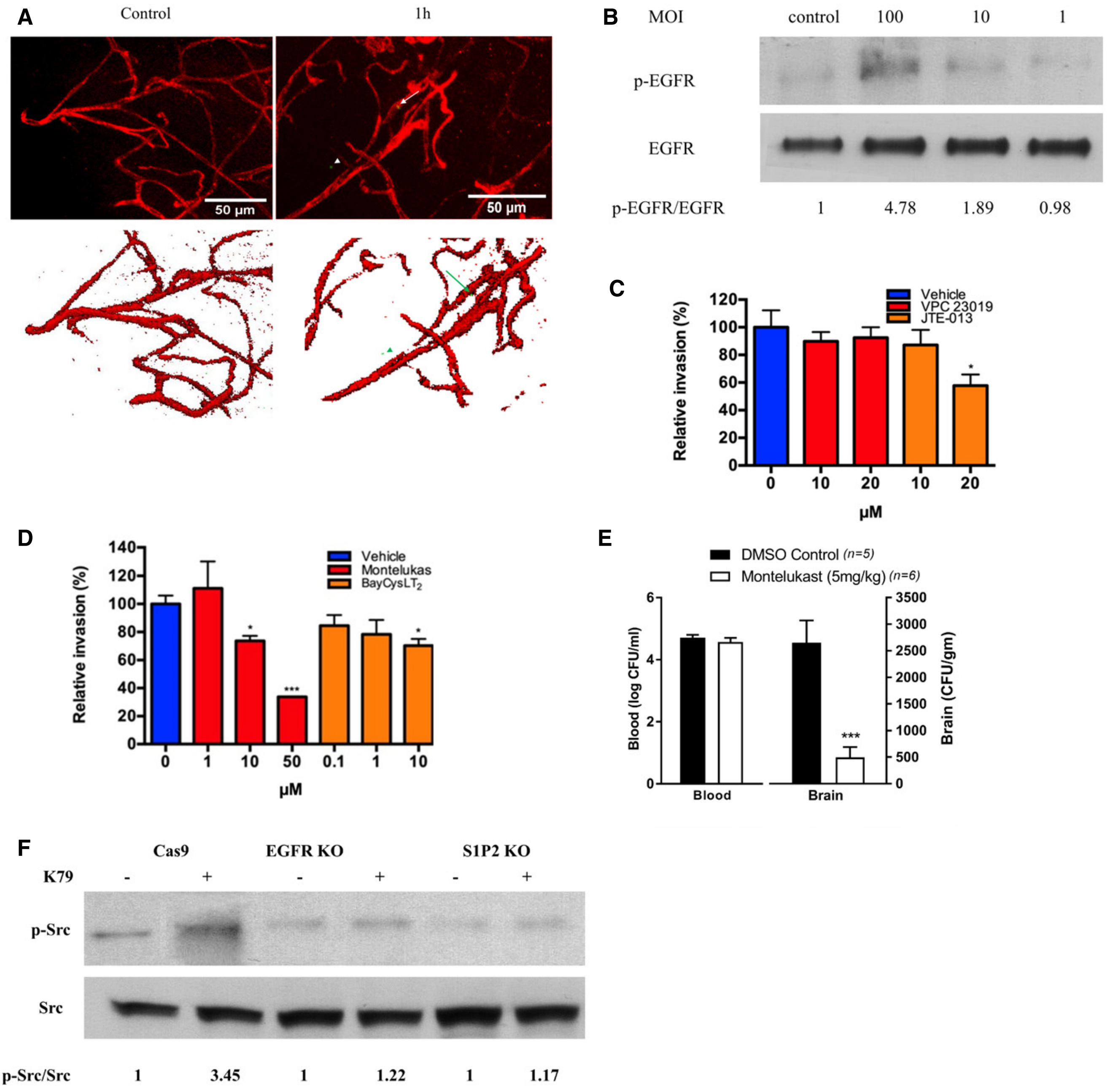

Figure EV3. GBS exploits host EGFR, S1P2 CysLT1 and Src for penetration of the blood-brain barrier.

A Wild-type mice received strain GFP-K79 via the tail vein. 1 h later, animals were perfused, and the cortex obtained for demonstration of claudin-5 (a marker for the tight junction proteins, for assessing structural integrity of the blood–brain barrier) during the GBS penetration. The arrows represent bacteria co-localized with capillaries, and arrowheads represent bacteria outside the capillaries. The result showed the intact tight junction one hour after infection. Scale bar = 50 μm.

B Tyrosine phosphorylation of EGFR in HBMEC in response to GBS strain K79 with MOI of 1, 10, and 100.

C GBS exploits host $S1P_2$ for invasion of HBMEC. Relative invasion frequency of strain K79 in HBMEC with or without $S1P_1/S1P_3$ antagonist (VPC23019) and $S1P_2$ antagonist (JTE-013). Data represent the means ± SEM of six independent experiments, with statistical analysis by Student's *t*-test, *P = 0.016.

D Relative invasion frequency of strain K79 in HBMEC with or without CysLT1 antagonist (montelukast) and CysLT2 antagonist (BayCysLT2). Data represent the means ± SEM of three independent experiments, with statistical analysis by Student's *t*-test. From left to right, *P = 0.020, ***P = 0.00038, *P = 0.018.

E Bacterial counts recovered from the blood and brain in wild-type mice receiving vehicle control or montelukast (5 mg/kg), infected with strain K79 for 1 h. Data represent the means ± SEM with statistical analysis by Wilcoxon rank sum test, ***P = 0.0043.

F c-Src was shown to be downstream of S1P2 and EGFR in GBS invasion of the BBB, as shown by the demonstration that c-Src phosphorylation in response to GBS was inhibited in EGFR and S1P2 KO cells.

Source data are available online for this figure.

 © 2021 The Authors

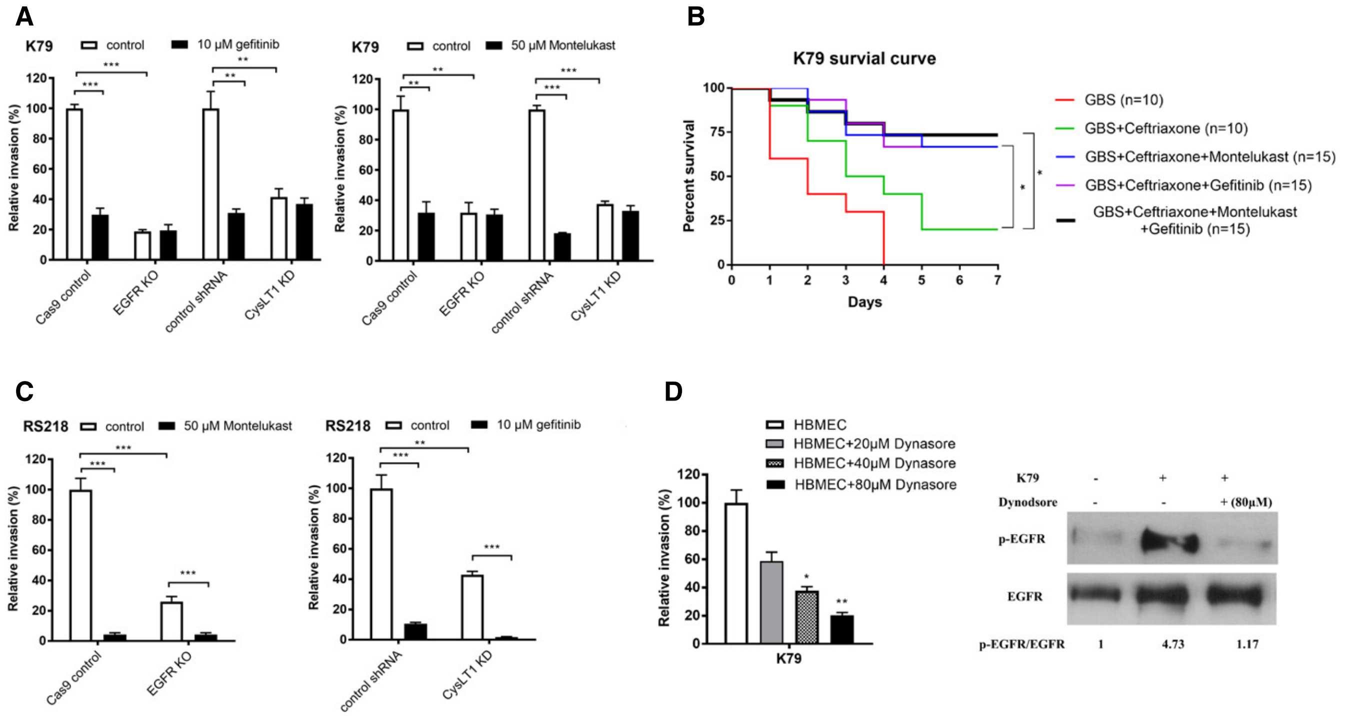

Figure EV4. Blocking EGFR and CysLT1 at the same time did not show additive effects of GBS invasion.

A Relative invasion frequency of GBS strain K79 in EGFR knockout and CysLT1 knockdown HBMEC with or without EGFR antagonist (gefitinib) and CysLT1 antagonist (montelukast). Data represent the means ± SEM of three independent experiments. In left panel, ****P* = 0.00016 between control and gefitinib in Cas9 control HBMEC, ***P* = 0.0038 between control and gefitinib in shRNA control HBMEC. In right panel, ***P* = 0.0037 between control and montelukast in Cas9 control HBMEC, ****P* = 6.452E-06 between control and montelukast in shRNA control HBMEC.

B GBS group *n* = 10, CFX group *n* = 10, CFX + MONT group *n* = 15, CFX + GEF group *n* = 15, CFX + GEF + MONT group *n* = 15. Data are presented as a Kaplan–Maier plot with a log-rank test. Survival was greater with ceftriaxone plus gefitinib (**P* = 0.017) or montelukast (**P* = 0.019) compared to ceftriaxone alone. However, administration of two inhibitors together did not provide additional improved survival compared to individual inhibitor.

C Relative invasion frequency of *E. coli* strain RS218 in EGFR knockout and CysLT1 knockdown HBMEC with or without CysLT1 antagonist (montelukast) and EGFR antagonist (gefitinib), respectively. Data represent the means ± SEM of three independent experiments. In left panel, ****P* = 0.00023 between control and montelukast group in Cas9 control HBMEC, ****P* = 0.00036 between control and montelukast group in EGFR knockout HBMEC. In right panel, ****P* = 0.00044 between control and gefitinib group in shRNA control HBMEC, ****P* = 0.00099 between control and gefitinib group in CysLT1 knockdown HBMEC.

D We showed that GBS invasion of HBMEC involves clathrin-mediated endocytosis and inhibition of clathrin-mediated endocytosis was effective in inhibition of GBS invasion. This concept is shown by dynasore (a cell-permeable inhibitor of clathrin-mediated endocytosis, Veiga *et al*, 2007), and dynasore inhibited GBS invasion of HBMEC. Invasion Data represent the means ± SEM of three independent experiments, **P* = 0.0028, ***P* = 0.00099. These findings support the role of endocytosis in GBS invasion of HBMEC. This concept is also supported by the demonstration that EGFR activation in response to GBS was inhibited by dynasore, supporting that inhibition of endocytosis results in inhibition of host cell signaling cascade involved in GBS invasion of HBMEC.

Source data are available online for this figure.
