# Supplementary material for: Therapeutic development of group B Streptococcus meningitis by targeting a host cell signaling network involving EGFR
Source: EMBO Mol Med. 2021 Jan 21;13(3):e12651. doi: 10.15252/emmm.202012651 (PMC7933950; doi:10.15252/emmm.202012651)

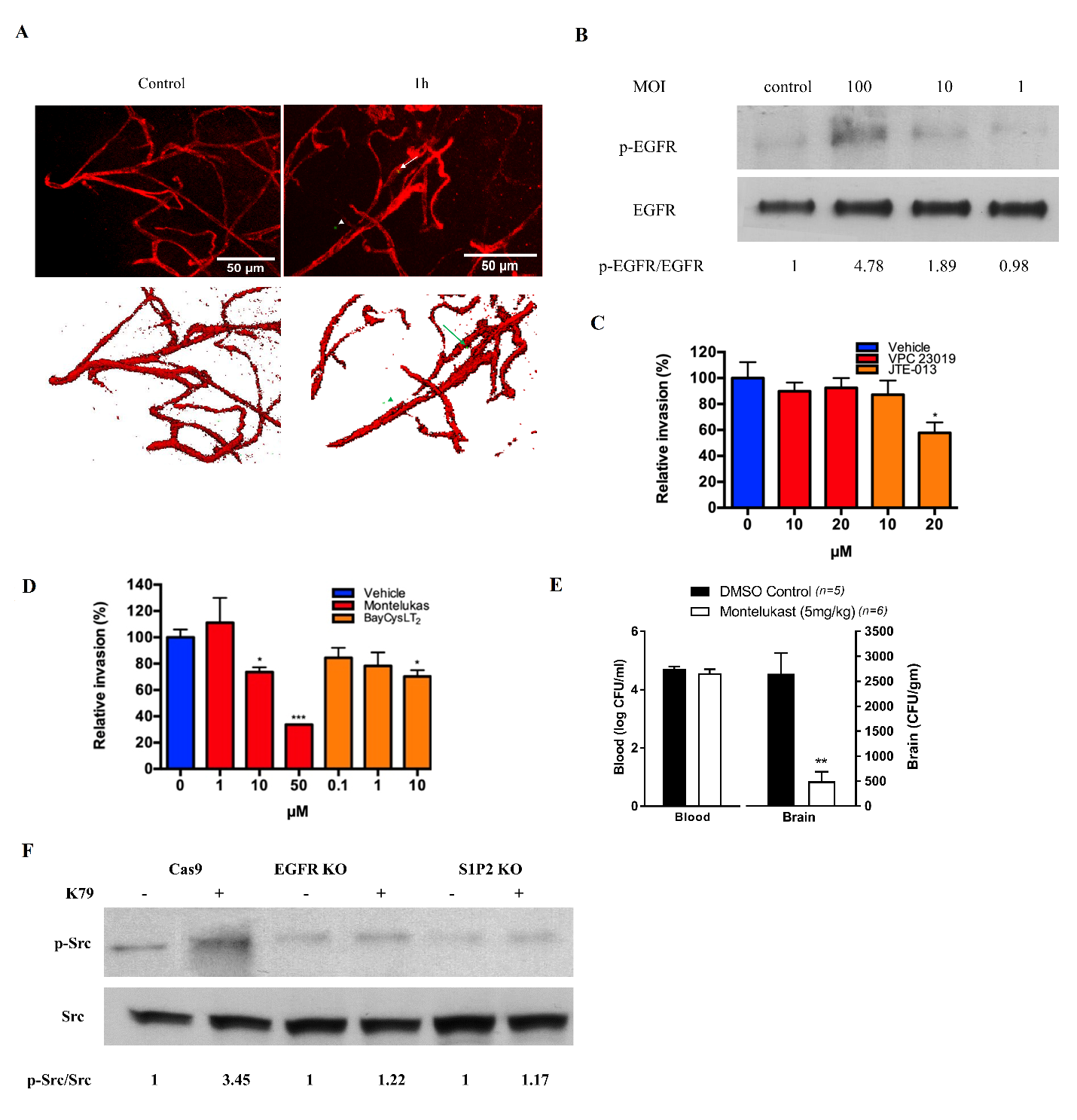


EV3B Tyrosine phosphorylation of EGFR in HBMEC in response to GBS strain K79 with MOI of 1, 10, and 100


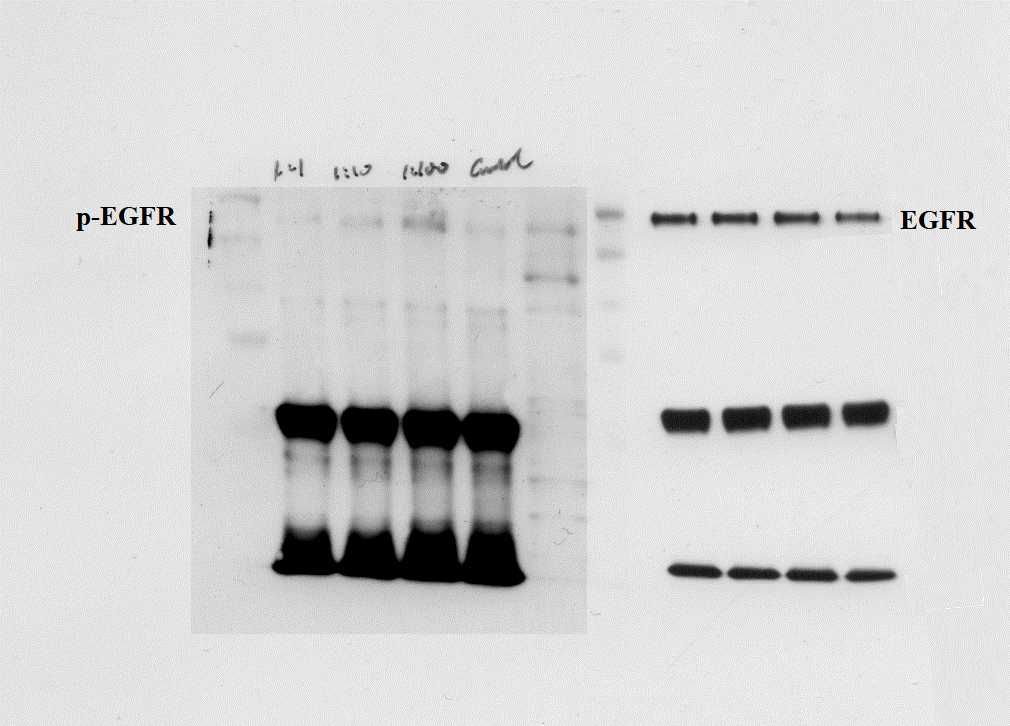


EV3C GBS exploits host S1P2 for invasion of HBMEC. Relative invasion frequency of strain K79 in HBMEC with or without S1P1/S1P3 antagonist (VPC23019) and S1P2 antagonist (JTE-013)

|  | Dilution | colonies | average | percentage | ave percentage | p value |
| --- | --- | --- | --- | --- | --- | --- |
| Control DMSO | 0 | 129 | 164.5 | 78.419453 | 100 |  |
| Control DMSO | 0 | 126 |  | 76.595745 |  |  |
| Control DMSO | 0 | 112 |  | 68.085106 |  |  |
| Control DMSO | 0 | 230 |  | 139.81763 |  |  |
| Control DMSO | 0 | 180 |  | 109.42249 |  |  |
| Control DMSO | 0 | 210 |  | 127.65957 |  |  |
| VPC 10 µM | 0 | 156 | 147.66667 | 94.832827 | 89.766971 | 0.4802949 |
| VPC 10 µM | 0 | 154 |  | 93.617021 |  |  |
| VPC 10 µM | 0 | 136 |  | 82.674772 |  |  |
| VPC 10 µM | 0 | 160 |  | 97.264438 |  |  |
| VPC 10 µM | 0 | 100 |  | 60.790274 |  |  |
| VPC 10 µM | 0 | 180 |  | 109.42249 |  |  |
| VPC 20 µM | 0 | 155 | 152 | 94.224924 | 92.401216 | 0.6089251 |
| VPC 20 µM | 0 | 168 |  | 102.12766 |  |  |
| VPC 20 µM | 0 | 109 |  | 66.261398 |  |  |
| VPC 20 µM | 0 | 180 |  | 109.42249 |  |  |
| VPC 20 µM | 0 | 180 |  | 109.42249 |  |  |
| VPC 20 µM | 0 | 120 |  | 72.948328 |  |  |
| JTE 10 µM | 0 | 195 | 143.33333 | 118.54103 | 87.132725 | 0.4514879 |
| JTE 10 µM | 0 | 126 |  | 76.595745 |  |  |
| JTE 10 µM | 0 | 119 |  | 72.340426 |  |  |
| JTE 10 µM | 0 | 200 |  | 121.58055 |  |  |
| JTE 10 µM | 0 | 130 |  | 79.027356 |  |  |
| JTE 10 µM | 0 | 90 |  | 54.711246 |  |  |
| JTE 20 µM | 0 | 86 | 95 | 52.279635 | 57.75076 | 0.0161156 |
| JTE 20 µM | 0 | 82 |  | 49.848024 |  |  |
| JTE 20 µM | 0 | 72 |  | 43.768997 |  |  |
| JTE 20 µM | 0 | 140 |  | 85.106383 |  |  |
| JTE 20 µM | 0 | 130 |  | 79.027356 |  |  |
| JTE 20 µM | 0 | 60 |  | 36.474164 |  |  |

EV3D Relative invasion frequency of strain K79 in HBMEC with or without CysLT1 antagonist (montelukast) and CysLT2 antagonist (BayCysLT2)

|  | Dilution | colonies | average | percentage | ave percentage | p value |
| --- | --- | --- | --- | --- | --- | --- |
| Control DMSO | 10 | 116 | 107.66667 | 107.73994 | 100 |  |
| Control DMSO | 10 | 112 |  | 104.02477 |  |  |
| Control DMSO | 10 | 95 |  | 88.235294 |  |  |
| Montelukast 1 µM | 10 | 140 | 119.66667 | 130.03096 | 111.14551 | 0.6037197 |
| Montelukast 1 µM | 10 | 140 |  | 130.03096 |  |  |
| Montelukast 1 µM | 10 | 79 |  | 73.374613 |  |  |
| Montelukast 10 µM | 10 | 74 | 79.333333 | 68.73065 | 73.684211 | 0.0198349 |
| Montelukast 10 µM | 10 | 87 |  | 80.804954 |  |  |
| Montelukast 10 µM | 10 | 77 |  | 71.517028 |  |  |
| Montelukast 50 µM | 10 | 37 | 36.333333 | 34.365325 | 33.74613 | 0.0003793 |
| Montelukast 50 µM | 10 | 36 |  | 33.436533 |  |  |
| Montelukast 50 µM | 10 | 36 |  | 33.436533 |  |  |
| BayCysLT2 0.1 µM | 10 | 102 | 91 | 94.736842 | 84.520124 | 0.1847488 |
| BayCysLT2 0.1 µM | 10 | 96 |  | 89.164087 |  |  |
| BayCysLT2 0.1 µM | 10 | 75 |  | 69.659443 |  |  |
| BayCysLT2 1 µM | 10 | 102 | 84.333333 | 94.736842 | 78.328173 | 0.1421321 |
| BayCysLT2 1 µM | 10 | 64 |  | 59.442724 |  |  |
| BayCysLT2 1 µM | 10 | 87 |  | 80.804954 |  |  |
| BayCysLT2 10 µM | 10 | 75 | 75.666667 | 69.659443 | 70.278638 | 0.018073 |
| BayCysLT2 10 µM | 10 | 67 |  | 62.229102 |  |  |
| BayCysLT2 10 µM | 10 | 85 |  | 78.947368 |  |  |

EV3E Bacterial counts recovered from the blood and brain in wild type mice receiving vehicle control or montelukast (5 mg/kg), infected with strain K79 for 1h.

| Mice | Bacteria | Blood | | Brain (100 µl/1000 µl) | |
| --- | --- | --- | --- | --- | --- |
|  |  | volume (ul) | CFU 10^-1^ | weight (g) | CFU 10^0^ |
| DMSO Control | K79 | 20 | 161 | 0.44 | 180 |
| DMSO Control | K79 | 20 | 175 | 0.42 | 91 |
| DMSO Control | K79 | 20 | 73 | 0.43 | 80 |
| DMSO Control | K79 | 20 | 61 | 0.47 | 146 |
| DMSO Control | K79 | 20 | 93 | 0.45 | 91 |
| Montelukast (5mg/kg) | K79 | 20 | 147 | 0.42 | 10 |
| Montelukast (5mg/kg) | K79 | 20 | 151 | 0.41 | 58 |
| Montelukast (5mg/kg) | K79 | 20 | 34 | 0.44 | 19 |
| Montelukast (5mg/kg) | K79 | 20 | 80 | 0.45 | 23 |
| Montelukast (5mg/kg) | K79 | 20 | 102 | 0.43 | 13 |
| Montelukast (5mg/kg) | K79 | 20 | 29 | 0.45 | 3 |

|  |  | Blood (CFU/ml) | Log10 | Brain (CFU/g) |
| --- | --- | --- | --- | --- |
| WT B6 | K79 | 80500 | 4.90579588 | 4090.909091 |
| WT B6 | K79 | 87500 | 4.94200805 | 2166.666667 |
| WT B6 | K79 | 36500 | 4.56229286 | 1860.465116 |
| WT B6 | K79 | 30500 | 4.48429984 | 3106.382979 |
| WT B6 | K79 | 46500 | 4.66745295 | 2022.222222 |
| Montelukast (5mg/kg) | K79 | 73500 | 4.86628734 | 238.0952381 |
| Montelukast (5mg/kg) | K79 | 75500 | 4.87794695 | 1414.634146 |
| Montelukast (5mg/kg) | K79 | 17000 | 4.23044892 | 431.8181818 |
| Montelukast (5mg/kg) | K79 | 40000 | 4.60205999 | 511.1111111 |
| Montelukast (5mg/kg) | K79 | 51000 | 4.70757018 | 302.3255814 |
| Montelukast (5mg/kg) | K79 | 14500 | 4.161368 | 66.66666667 |
| p value |  |  |  | 0.0043 |

EV3F c-Src was shown to be downstream of S1P2 and EGFR in GBS invasion of the BBB, as shown by the demonstration that c-Src phosphorylation in response to GBS was inhibited in EGFR and S1P2 KO cells


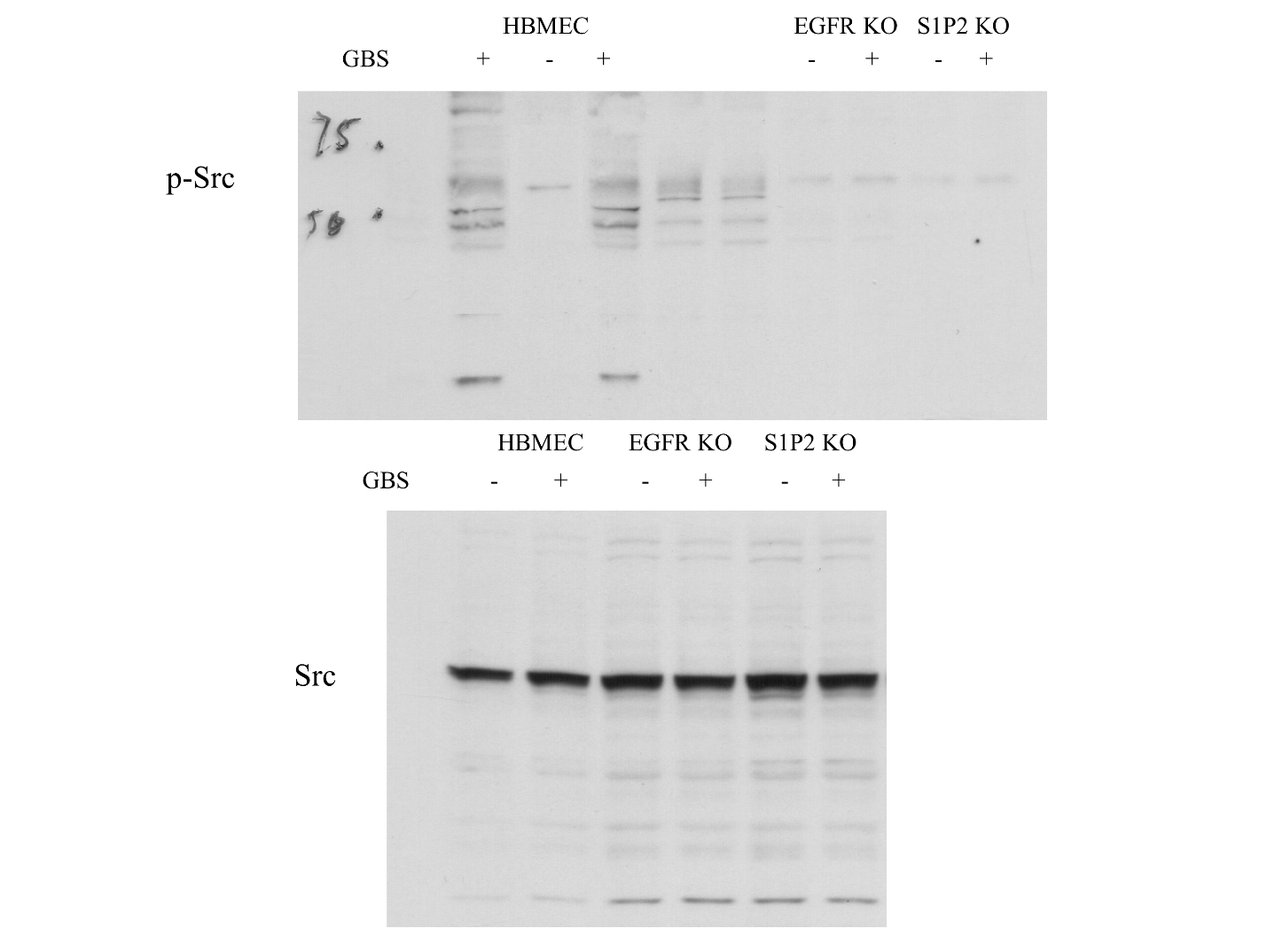

Supplement: Supplementary file 3 — Source Data for Expanded View and Appendix [file EMMM-13-e12651-s007.zip › Source_data_EV3.docx]
