# Supplementary material for: Therapeutic development of group B Streptococcus meningitis by targeting a host cell signaling network involving EGFR
Source: EMBO Mol Med. 2021 Jan 21;13(3):e12651. doi: 10.15252/emmm.202012651 (PMC7933950; doi:10.15252/emmm.202012651)

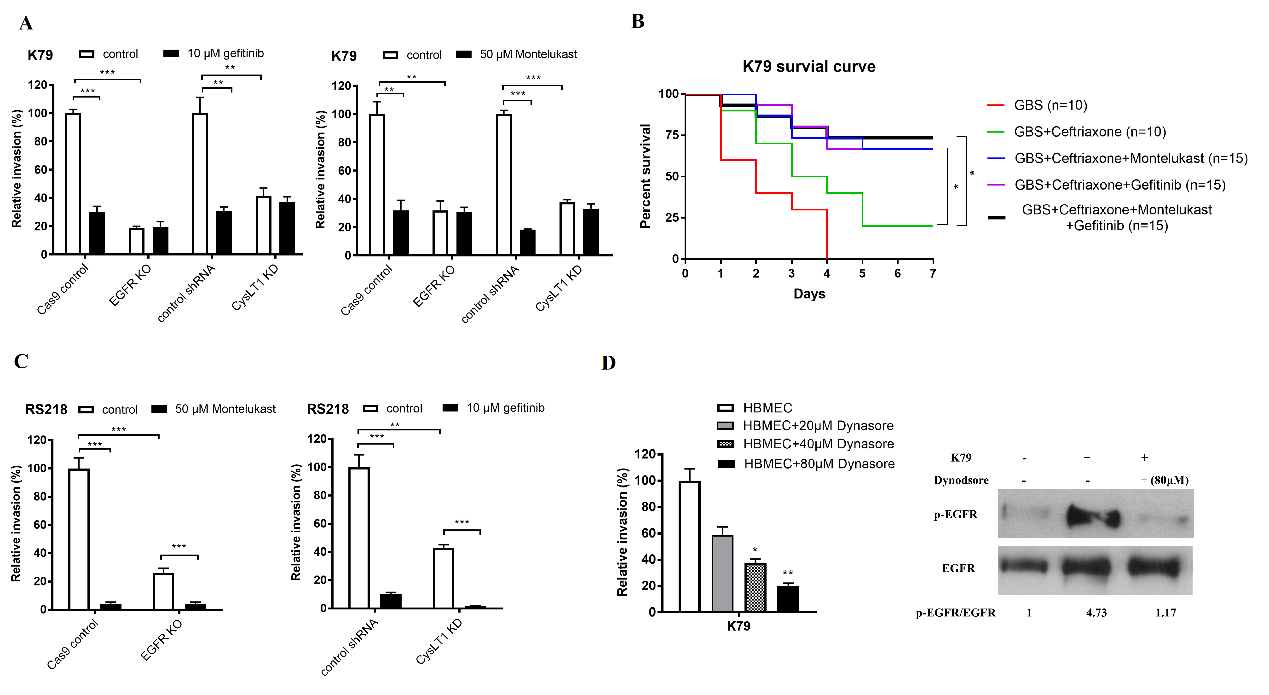


EV4A (A) Relative invasion frequency of GBS strain K79 in EGFR knockout and CysLT1 knockdown HBMEC with or without EGFR antagonist (gefitinib) and CysLT1 antagonist (montelukast).

| K79 |  | control |  |  | 10 μM Gefitinib |  | p value |
| --- | --- | --- | --- | --- | --- | --- | --- |
| Cas9 | 146 | 152 | 139 | 47 | 52 | 31 | 0.0001555 |
| EGFR KO | 28 | 30 | 24 | 28 | 19 | 38 | 0.8706887 |
| K79 |  | control |  |  | 10 μM Gefitinib |  | p value |
| Control shRNA | 325 | 220 | 295 | 100 | 85 | 75 | 0.0038109 |
| CysLT1 | 144 | 113 | 91 | 125 | 95 | 90 | 0.5386653 |

| K79 |  | control |  |  | 50 μM Montelukast |  | p value |
| --- | --- | --- | --- | --- | --- | --- | --- |
| Cas9 | 61 | 45 | 54 | 11 | 24 | 16 | 0.0037111 |
| EGFR KO | 14 | 13 | 24 | 20 | 14 | 15 | 0.8748561 |
| K79 |  | control |  |  | 50 μM Montelukast |  | p value |
| Control shRNA | 144 | 141 | 132 | 24 | 26 | 26 | 6.452E-06 |
| CysLT1 | 49 | 51 | 57 | 53 | 48 | 37 | 0.2982627 |

EV4B Survive Curve with Montelukast and Gefitinib

| Survive Curve EV | | |  |  |  |
| --- | --- | --- | --- | --- | --- |
|  | GBS (n=10) | GBS+CFX (n=10) | GBS+CFX+MON (n=15) | GBS+CFX+GEF (n=15) | GBS+CEF+MON+GEF (n=15) |
| Day1 | 6 | 9 | 15 | 15 | 14 |
| Day2 | 4 | 7 | 13 | 14 | 13 |
| Day3 | 3 | 5 | 11 | 12 | 12 |
| Day4 | 0 | 4 | 11 | 10 | 11 |
| Day5 | 0 | 2 | 10 | 10 | 11 |
| Day6 | 0 | 2 | 10 | 10 | 11 |
| Day7 | 0 | 2 | 10 | 10 | 11 |
| p value |  |  | 0.019 | 0.017 |  |

EV4C

| RS218 |  |  |  |  |  |  |  |
| --- | --- | --- | --- | --- | --- | --- | --- |
| -1 |  | control |  |  | 10 μM Gefitinib |  | p value |
| GFP | 84 | 114 | 96 | 9 | 12 | 10 | 0.000560919 |
| CysLT1 | 46 | 42 | 38 | 2 | 2 | 1 | 6.57315E-05 |
| p value | 0.0034225 |  |  |  |  |  |  |
|  |  |  |  |  |  |  |  |
| RS218 |  |  |  |  |  |  |  |
| -1 |  | control |  |  | 10 μM Gefitinib | | p value |
| Cas9 | 70 | 68 | 81 | 22 | 10 | 12 | 0.000443345 |
| EGFR KO | 28 | 29 | 32 | 12 | 17 | 13 | 0.000986526 |
| p value | 0.0005055 |  |  |  |  |  |  |

EV4D

|  | Dilution | colonies | average | percentage | ave percentage | p value |
| --- | --- | --- | --- | --- | --- | --- |
| HBMEC | 10 | 48 | 46 | 104.34783 | 100 |  |
| HBMEC | 10 | 52 |  | 113.04348 |  |  |
| HBMEC | 10 | 38 |  | 82.608696 |  |  |
| HBMEC + 20 uM dynosore | 10 | 39 | 31 | 84.782609 | 67 | 0.0808 |
| HBMEC + 20 uM dynosore | 10 | 32 |  | 69.565217 |  |  |
| HBMEC + 20 uM dynosore | 10 | 22 |  | 47.826087 |  |  |
| HBMEC + 40 uM dynosore | 10 | 20 | 17.333333 | 43.478261 | 38 | 0.002797 |
| HBMEC + 40 uM dynosore | 10 | 16 |  | 34.782609 |  |  |
| HBMEC + 40 uM dynosore | 10 | 16 |  | 34.782609 |  |  |
| HBMEC + 100 uM dynosore | 10 | 9 | 9.3333333 | 19.565217 | 20 | 0.000997 |
| HBMEC + 100 uM dynosore | 10 | 11 |  | 23.913043 |  |  |
| HBMEC + 100 uM dynosore | 10 | 8 |  | 17.391304 |  |  |


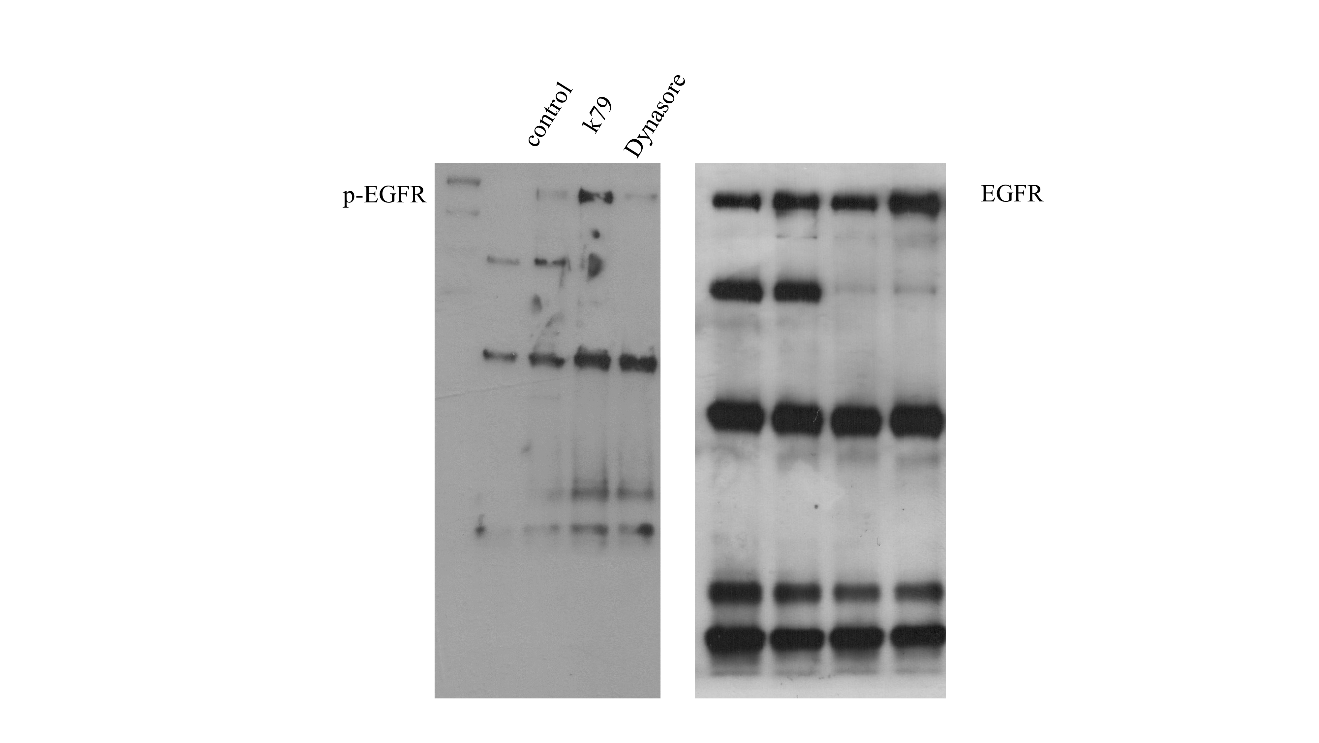

Supplement: Supplementary file 3 — Source Data for Expanded View and Appendix [file EMMM-13-e12651-s007.zip › Source_data_EV4.docx]
