# Supplementary material for: Therapeutic development of group B Streptococcus meningitis by targeting a host cell signaling network involving EGFR
Source: EMBO Mol Med. 2021 Jan 21;13(3):e12651. doi: 10.15252/emmm.202012651 (PMC7933950; doi:10.15252/emmm.202012651)
Supplement: Supplementary file 5 — Source Data for Figure 1 [file EMMM-13-e12651-s003.docx]

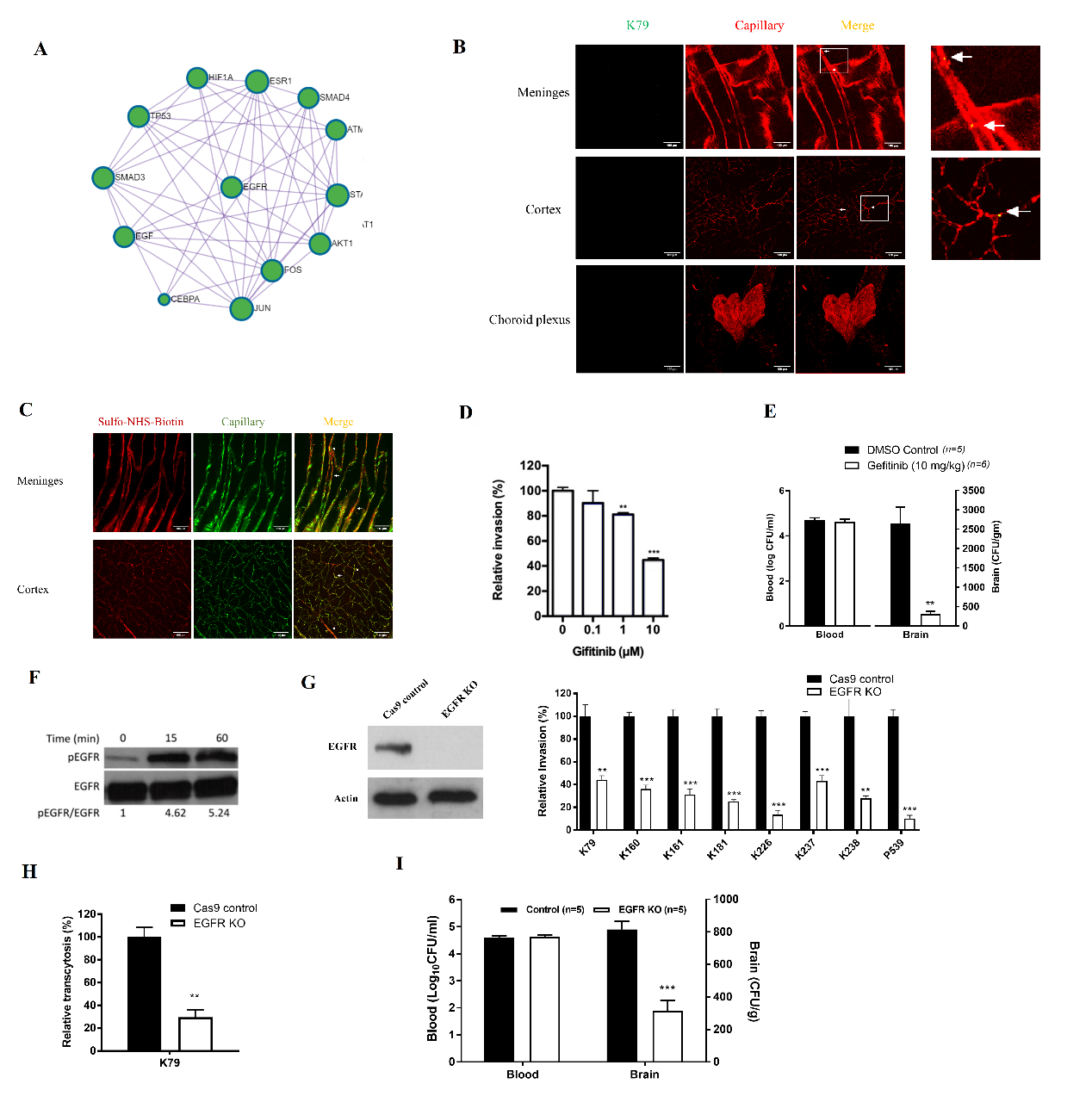


1D Relative invasion frequency of GBS strain K79 in HBMEC with or without EGFR inhibitor (gefitinib)

| K79 gifitinib invasion |  |  |  |  |  |  |
| --- | --- | --- | --- | --- | --- | --- |
|  | Dilution | colonies | average | percentage | ave percentage | p value |
| HBMEC | 100 | 59 | 60.666667 | 97.25275 | 100 |  |
| HBMEC | 100 | 61 |  | 100.5495 |  |  |
| HBMEC | 100 | 62 |  | 102.1978 |  |  |
| HBMEC + 10μM gifitinib | 100 | 49 | 55.333333 | 80.76923 | 91 | 0.181347 |
| HBMEC + 10μM gifitinib | 100 | 58 |  | 95.6044 |  |  |
| HBMEC + 10μM gifitinib | 100 | 59 |  | 97.25275 |  |  |
| HBMEC + 10μM gifitinib | 100 | 47 | 48 | 77.47253 | 79 | 0.001991 |
| HBMEC + 10μM gifitinib | 100 | 46 |  | 75.82418 |  |  |
| HBMEC + 10μM gifitinib | 100 | 51 |  | 84.06593 |  |  |
| HBMEC + 10μM gifitinib | 100 | 26 | 24.666667 | 42.85714 | 41 | 8.58E-06 |
| HBMEC + 10μM gifitinib | 100 | 23 |  | 37.91209 |  |  |
| HBMEC + 10μM gifitinib | 100 | 25 |  | 41.20879 |  |  |

1E Bacterial counts recovered from the blood and brain in wild type mice receiving vehicle control (n=5) or gefitinib (10 mg/kg) (n=6), infected with strain K79 for 1 h

| Mice | Bacteria | Blood | | Brain (100 µl/1000 µl) | |
| --- | --- | --- | --- | --- | --- |
|  |  | volume (ul) | CFU 10^-1^ | weight (g) | CFU 10^0^ |
| DMSO Control | K79 | 20 | 161 | 0.44 | 180 |
| DMSO Control | K79 | 20 | 175 | 0.42 | 91 |
| DMSO Control | K79 | 20 | 73 | 0.43 | 80 |
| DMSO Control | K79 | 20 | 61 | 0.47 | 146 |
| DMSO Control | K79 | 20 | 93 | 0.45 | 91 |
| Gefitinib (10 mg/kg) | K79 | 20 | 120 | 0.47 | 7 |
| Gefitinib (10 mg/kg) | K79 | 20 | 35 | 0.45 | 11 |
| Gefitinib (10 mg/kg) | K79 | 20 | 48 | 0.44 | 28 |
| Gefitinib (10 mg/kg) | K79 | 20 | 160 | 0.43 | 9 |
| Gefitinib (10 mg/kg) | K79 | 20 | 122 | 0.46 | 18 |
| Gefitinib (10 mg/kg) | K79 | 20 | 97 | 0.45 | 10 |

|  |  | Blood (CFU/ml) | Log10 | Brain (CFU/g) |
| --- | --- | --- | --- | --- |
| WT B6 | K79 | 80500 | 4.90579588 | 4090.9091 |
| WT B6 | K79 | 87500 | 4.942008053 | 2166.6667 |
| WT B6 | K79 | 36500 | 4.562292864 | 1860.4651 |
| WT B6 | K79 | 30500 | 4.484299839 | 3106.3830 |
| WT B6 | K79 | 46500 | 4.667452953 | 2022.2222 |
| Gefitinib (10 mg/kg) | K79 | 60000 | 4.77815125 | 148.9362 |
| Gefitinib (10 mg/kg) | K79 | 17500 | 4.243038049 | 244.4444 |
| Gefitinib (10 mg/kg) | K79 | 24000 | 4.380211242 | 636.3636 |
| Gefitinib (10 mg/kg) | K79 | 80000 | 4.903089987 | 209.3023 |
| Gefitinib (10 mg/kg) | K79 | 61000 | 4.785329835 | 391.3043 |
| Gefitinib (10 mg/kg) | K79 | 48500 | 4.685741739 | 222.2222 |
| p value |  |  |  | 0.0038 |

1G EGFR protein expression in EGFR knockout HBMEC using CRISPR/Cas9 and relative invasion frequency of 8 meningitis isolates of GBS strains in EGFR knockout and control HBMEC.


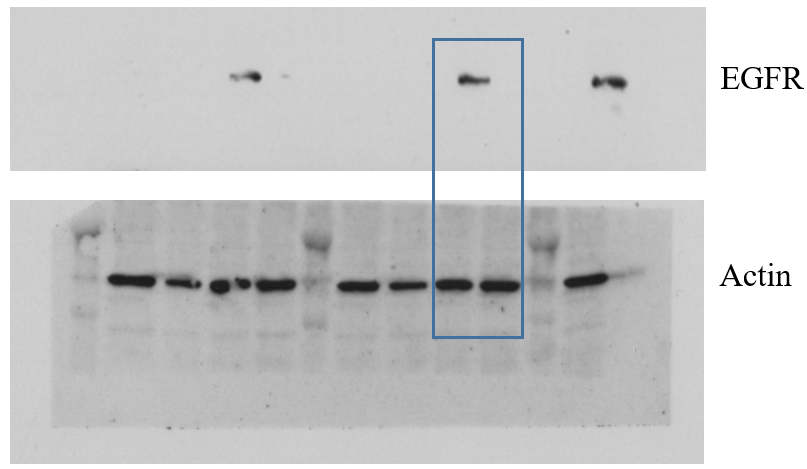


|  | **K79** |  |  |  |  |  |
| --- | --- | --- | --- | --- | --- | --- |
| Cells | dilution | # of colonies | Average | percentage | ave percentage | pValue |
| Cas9 | 100 | 41 | 35.333333 | 116 | 100 | 0.0059189 |
| Cas9 | 100 | 29 |  | 82 |  |  |
| Cas9 | 100 | 36 |  | 102 |  |  |
| EGFR KO | 100 | 18 | 15.666667 | 51 | 44 |  |
| EGFR KO | 100 | 14 |  | 40 |  |  |
| EGFR KO | 100 | 15 |  | 42 |  |  |
|  |  |  |  |  |  |  |
|  |  |  |  |  |  |  |
|  | **160** |  |  |  |  |  |
| Genes | dilution | # of colonies | Average | percentage | ave percentage | pValue |
| Cas9 | 10 | 194 | 188.66667 | 103 | 100 | 0.0002448 |
| Cas9 | 10 | 175 |  | 93 |  |  |
| Cas9 | 10 | 197 |  | 104 |  |  |
| EGFR KO | 10 | 67 | 67.666667 | 36 | 36 |  |
| EGFR KO | 10 | 56 |  | 30 |  |  |
| EGFR KO | 10 | 80 |  | 42 |  |  |
|  |  |  |  |  |  |  |
|  |  |  |  |  |  |  |
|  | **161** |  |  |  |  |  |
| Genes | dilution | # of colonies | Average | percentage | ave percentage | pValue |
| Cas9 | 10 | 132 | 148 | 89 | 100 | 0.0008041 |
| Cas9 | 10 | 161 |  | 109 |  |  |
| Cas9 | 10 | 151 |  | 102 |  |  |
| EGFR KO | 10 | 59 | 46.333333 | 40 | 31 |  |
| EGFR KO | 10 | 46 |  | 31 |  |  |
| EGFR KO | 10 | 34 |  | 23 |  |  |
|  |  |  |  |  |  |  |
|  |  |  |  |  |  |  |
|  | **181** |  |  |  |  |  |
| Genes | dilution | # of colonies | Average | percentage | ave percentage | pValue |
| Cas9 | 10 | 104 | 119.66667 | 87 | 100 | 0.0004246 |
| Cas9 | 10 | 131 |  | 109 |  |  |
| Cas9 | 10 | 124 |  | 104 |  |  |
| EGFR KO | 10 | 34 | 30.666667 | 28 | 26 |  |
| EGFR KO | 10 | 28 |  | 23 |  |  |
| EGFR KO | 10 | 30 |  | 25 |  |  |
|  |  |  |  |  |  |  |
|  | **226** |  |  |  |  |  |
| Genes | dilution | # of colonies | Average | percentage | ave percentage | pValue |
| Cas9 | 100 | 21 | 19.333333 | 109 | 100 | 0.0001128 |
| Cas9 | 100 | 18 |  | 93 |  |  |
| Cas9 | 100 | 19 |  | 98 |  |  |
| EGFR KO | 100 | 4 | 2.6666667 | 21 | 14 |  |
| EGFR KO | 100 | 2 |  | 10 |  |  |
| EGFR KO | 100 | 2 |  | 10 |  |  |
|  |  |  |  |  |  |  |
|  | **237** |  |  |  |  |  |
| Genes | dilution | # of colonies | Average | percentage | ave percentage | pValue |
| Cas9 | 10 | 90 | 83.333333 | 108 | 100 | 0.0007186 |
| Cas9 | 10 | 82 |  | 98 |  |  |
| Cas9 | 10 | 78 |  | 94 |  |  |
| EGFR KO | 10 | 34 | 36 | 41 | 43 |  |
| EGFR KO | 10 | 31 |  | 37 |  |  |
| EGFR KO | 10 | 43 |  | 52 |  |  |
|  |  |  |  |  |  |  |
|  | **238** |  |  |  |  |  |
| Genes | dilution | # of colonies | Average | percentage | ave percentage | pValue |
| Cas9 | 100 | 14 | 19 | 74 | 100 | 0.0092876 |
| Cas9 | 100 | 19 |  | 100 |  |  |
| Cas9 | 100 | 24 |  | 126 |  |  |
| EGFR KO | 100 | 5 | 5.3333333 | 26 | 28 |  |
| EGFR KO | 100 | 6 |  | 32 |  |  |
| EGFR KO | 100 | 5 |  | 26 |  |  |
|  |  |  |  |  |  |  |
|  | **P539** |  |  |  |  |  |
| Genes | dilution | # of colonies | Average | percentage | ave percentage | pValue |
| Cas9 | 100 | 42 | 42 | 100 | 100 | 0.0001327 |
| Cas9 | 100 | 38 |  | 90 |  |  |
| Cas9 | 100 | 46 |  | 110 |  |  |
| EGFR KO | 100 | 6 |  | 14 | 10 |  |
| EGFR KO | 100 | 2 |  | 5 |  |  |
| EGFR KO | 100 | 5 |  | 12 |  |  |

1H GBS strain K79 traversal of HBMEC monolayer was significantly decreased in EGFR knockout HBMEC compare to control HBMEC

|  | Dilution | colonies | average | percentage | ave percentage | p value |
| --- | --- | --- | --- | --- | --- | --- |
| Cas9#14 | 10 | 265 | 301.6666667 | 87.845304 | 100 |  |
| Cas9#14 | 10 | 350 |  | 116.0221 |  |  |
| Cas9#14 | 10 | 290 |  | 96.132597 |  |  |
| EGFR KO | 10 | 94 | 89.33333333 | 31.160221 | 30 | 0.0026909 |
| EGFR KO | 10 | 53 |  | 17.569061 |  |  |
| EGFR KO | 10 | 121 |  | 40.110497 |  |  |

1I

Bacterial counts recovered from the blood and brain of EGFR conditional knockout (n=5) and control mice (n=5) 1 h after intravenous inoculation with strain K79.

| Mice | Bacteria | Blood | | Brain (100 µl/1000 µl) | |
| --- | --- | --- | --- | --- | --- |
|  |  | volume (ul) | CFU 10^-1^ | weight (g) | CFU 10^0^ |
| WT B6 | K79 | 20 | 103 | 0.46 | 44 |
| WT B6 | K79 | 20 | 76 | 0.46 | 40 |
| WT B6 | K79 | 20 | 48 | 0.45 | 38 |
| WT B6 | K79 | 20 | 87 | 0.48 | 34 |
| WT B6 | K79 | 20 | 105 | 0.46 | 32 |
| EGFR KO | K79 | 20 | 59 | 0.47 | 20 |
| EGFR KO | K79 | 20 | 67 | 0.47 | 6 |
| EGFR KO | K79 | 20 | 116 | 0.45 | 16 |
| EGFR KO | K79 | 20 | 105 | 0.48 | 22 |
| EGFR KO | K79 | 20 | 100 | 0.47 | 10 |

| Mice | Bacteria | Blood (CFU/ml) | Log10 | Brain (CFU/g) |
| --- | --- | --- | --- | --- |
| WT B6 | K79 | 51500 | 4.7118072 | 956.52174 |
| WT B6 | K79 | 38000 | 4.5797836 | 869.56522 |
| WT B6 | K79 | 24000 | 4.3802112 | 844.44444 |
| WT B6 | K79 | 43500 | 4.6384893 | 708.33333 |
| WT B6 | K79 | 52500 | 4.7201593 | 695.65217 |
| EGFR KO | K79 | 29500 | 4.469822 | 425.53191 |
| EGFR KO | K79 | 33500 | 4.5250448 | 127.65957 |
| EGFR KO | K79 | 58000 | 4.763428 | 355.55556 |
| EGFR KO | K79 | 52500 | 4.7201593 | 458.33333 |
| EGFR KO | K79 | 50000 | 4.69897 | 212.76596 |
| p value |  |  |  | 0.0002589 |
