# Supplementary material for: Therapeutic development of group B Streptococcus meningitis by targeting a host cell signaling network involving EGFR
Source: EMBO Mol Med. 2021 Jan 21;13(3):e12651. doi: 10.15252/emmm.202012651 (PMC7933950; doi:10.15252/emmm.202012651)
Supplement: Supplementary file 6 — Source Data for Figure 2 [file EMMM-13-e12651-s004.docx]

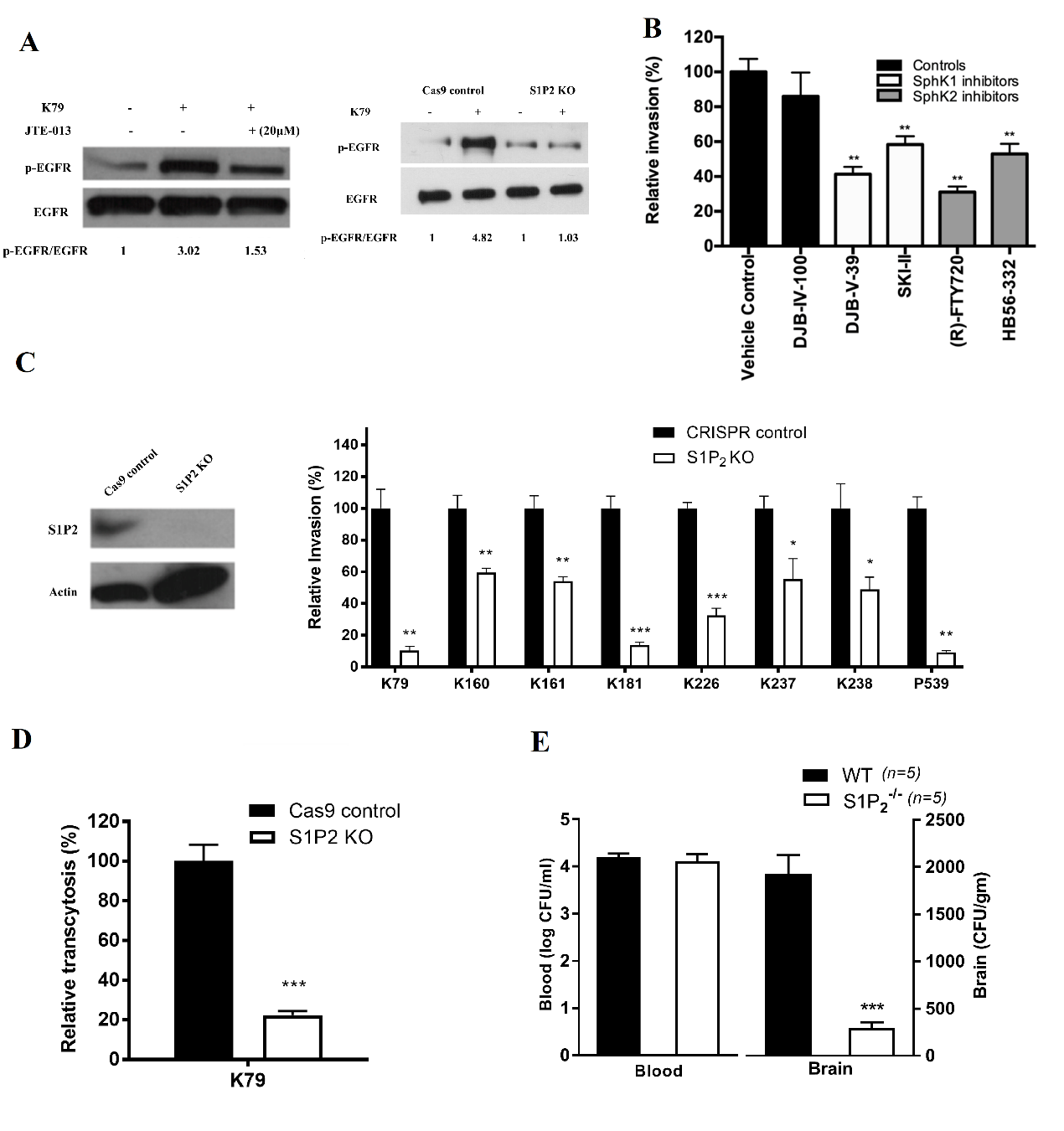


2A EGFR activation in S1P2 knockout HBMEC


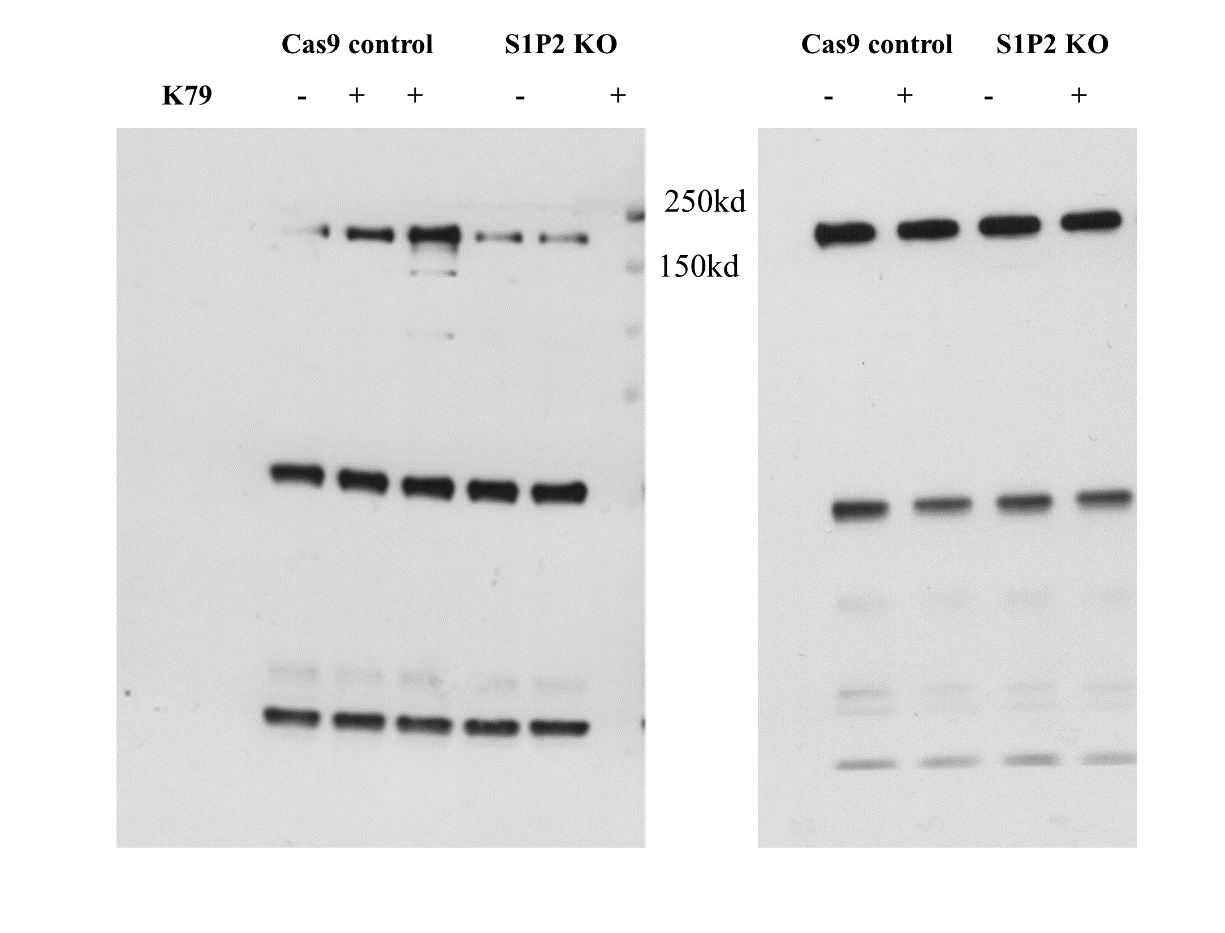


2B Relative invasion frequency of strain K79 in HBMEC with or without SphK1 and SphK2 inhibitors.

|  | Dilution | colonies | final | average | percentage | ave percentage | p value |
| --- | --- | --- | --- | --- | --- | --- | --- |
| DMSO control | 10 | 19 | 190 | 223.33333 | 85.074627 | 100 |  |
| DMSO control | 10 | 24 | 240 |  | 107.46269 |  |  |
| DMSO control | 10 | 24 | 240 |  | 107.46269 |  |  |
| R-FTY720 methyl ether | 0 | 81 | 81 | 69.333333 | 36.268657 | 31 | 0.0010647 |
| R-FTY721 methyl ether | 0 | 56 | 56 |  | 25.074627 |  |  |
| R-FTY722 methyl ether | 0 | 71 | 71 |  | 31.791045 |  |  |
| HB56-332 | 0 | 108 | 108 | 118.33333 | 48.358209 | 53 | 0.0075975 |
| HB56-332 | 0 | 103 | 103 |  | 46.119403 |  |  |
| HB56-332 | 0 | 144 | 144 |  | 64.477612 |  |  |
| DJB-IV-100 | 10 | 25 | 250 | 193.33333 | 111.9403 | 87 | 0.4273397 |
| DJB-IV-100 | 10 | 18 | 180 |  | 80.597015 |  |  |
| DJB-IV-100 | 10 | 15 | 150 |  | 67.164179 |  |  |
| DJB-V-39 | 0 | 85 | 85 | 92.333333 | 38.059701 | 41 | 0.0023832 |
| DJB-V-39 | 0 | 111 | 111 |  | 49.701493 |  |  |
| DJB-V-39 | 0 | 81 | 81 |  | 36.268657 |  |  |
| SKI-II | 0 | 151 | 151 | 130.33333 | 67.61194 | 58 | 0.0092403 |
| SKI-II | 0 | 124 | 124 |  | 55.522388 |  |  |
| SKI-II | 0 | 116 | 116 |  | 51.940299 |  |  |

2C S1P_2_ protein expression in S1P_2_ knockout HBMEC using CRISPR/Cas9 (left panel). Relative invasion frequency of 8 meningitis isolates of GBS in S1P_2_ knockout HBMEC

| Cells | dilution | # of colonies | Average | GBS strains | percentage | percent. Ave. | p value |
| --- | --- | --- | --- | --- | --- | --- | --- |
| Cas9 | 100 | 35 | 45.66666667 | K79 | 76.642336 | 100 | 0.0018365 |
| Cas9 | 100 | 53 |  | K79 | 116.05839 |  |  |
| Cas9 | 100 | 49 |  | K79 | 107.29927 |  |  |
| S1P2 | 100 | 3 | 4.666666667 | K79 | 6.5693431 | 10.218978 |  |
| S1P2 | 100 | 7 |  | K79 | 15.328467 |  |  |
| S1P2 | 100 | 4 |  | K79 | 8.7591241 |  |  |
|  |  |  |  |  |  |  |  |
| Cas9 | 100 | 105 | 118.3333333 | K160 | 88.732394 | 100 | 0.0091412 |
| Cas9 | 100 | 137 |  | K160 | 115.77465 |  |  |
| Cas9 | 100 | 113 |  | K160 | 95.492958 |  |  |
| S1P2 | 100 | 72 | 70.33333333 | K160 | 60.84507 | 59.43662 |  |
| S1P2 | 100 | 75 |  | K160 | 63.380282 |  |  |
| S1P2 | 100 | 64 |  | K160 | 54.084507 |  |  |
|  |  |  |  |  |  |  |  |
| Cas9 | 100 | 39 | 44.33333333 | K161 | 87.969925 | 100 | 0.0054059 |
| Cas9 | 100 | 51 |  | K161 | 115.03759 |  |  |
| Cas9 | 100 | 43 |  | K161 | 96.992481 |  |  |
| S1P2 | 100 | 22 | 24 | K161 | 49.62406 | 54.135338 |  |
| S1P2 | 100 | 24 |  | K161 | 54.135338 |  |  |
| S1P2 | 100 | 26 |  | K161 | 58.646617 |  |  |
|  |  |  |  |  |  |  |  |
| Cas9 | 10 | 36 | 41.66666667 | K181 | 86.4 | 100 | 0.0004008 |
| Cas9 | 10 | 47 |  | K181 | 112.8 |  |  |
| Cas9 | 10 | 42 |  | K181 | 100.8 |  |  |
| S1P2 | 10 | 4 | 5.666666667 | K181 | 9.6 | 13.6 |  |
| S1P2 | 10 | 7 |  | K181 | 16.8 |  |  |
| S1P2 | 10 | 6 |  | K181 | 14.4 |  |  |
|  |  |  |  |  |  |  |  |
| Cas9 | 100 | 39 | 41 | K226 | 95.121951 | 100 | 0.0003253 |
| Cas9 | 100 | 40 |  | K226 | 97.560976 |  |  |
| Cas9 | 100 | 44 |  | K226 | 107.31707 |  |  |
| S1P2 | 100 | 17 | 13.33333333 | K226 | 41.463415 | 32.520325 |  |
| S1P2 | 100 | 12 |  | K226 | 29.268293 |  |  |
| S1P2 | 100 | 11 |  | K226 | 26.829268 |  |  |
|  |  |  |  |  |  |  |  |
| Cas9 | 100 | 57 | 50 | K237 | 114 | 100 | 0.0403239 |
| Cas9 | 100 | 49 |  | K237 | 98 |  |  |
| Cas9 | 100 | 44 |  | K237 | 88 |  |  |
| S1P2 | 100 | 36 | 27.66666667 | K237 | 72 | 55.333333 |  |
| S1P2 | 100 | 15 |  | K237 | 30 |  |  |
| S1P2 | 100 | 32 |  | K237 | 64 |  |  |
|  |  |  |  |  |  |  |  |
| Cas9 | 10 | 321 | 259.6666667 | K238 | 123.62003 | 100 | 0.0407682 |
| Cas9 | 10 | 185 |  | K238 | 71.245186 |  |  |
| Cas9 | 10 | 273 |  | K238 | 105.13479 |  |  |
| S1P2 | 10 | 136 | 126.6666667 | K238 | 52.37484 | 48.780488 |  |
| S1P2 | 10 | 88 |  | K238 | 33.889602 |  |  |
| S1P2 | 10 | 156 |  | K238 | 60.077022 |  |  |
|  |  |  |  |  |  |  |  |
| Cas9 | 10 | 274 | 240.6666667 | P539 | 113.85042 | 100 | 0.0002538 |
| Cas9 | 10 | 234 |  | P539 | 97.229917 |  |  |
| Cas9 | 10 | 214 |  | P539 | 88.919668 |  |  |
| S1P2 | 10 | 27 | 22.33333333 | P539 | 11.218837 | 9.2797784 |  |
| S1P2 | 10 | 21 |  | P539 | 8.7257618 |  |  |
| S1P2 | 10 | 19 |  | P539 | 7.8947368 |  |  |

2D GBS strain K79 traversal of HBMEC monolayer was significantly decreased in S1P2 knockout HBMEC compare to control

|  | Dilution | colonies | average | percentage | ave percentage | p value |
| --- | --- | --- | --- | --- | --- | --- |
| Cas9#14 | 10 | 265 | 301.66667 | 87.845304 | 100 |  |
| Cas9#14 | 10 | 350 |  | 116.0221 |  |  |
| Cas9#14 | 10 | 290 |  | 96.132597 |  |  |
| S1P2 | 10 | 58 | 67.333333 | 19.226519 | 22 | 0.000835 |
| S1P2 | 10 | 65 |  | 21.546961 |  |  |
| S1P2 | 10 | 79 |  | 26.187845 |  |  |

2E Bacterial counts recovered from the blood and brain of wild type and S1P_2_ ^-/-^ mice.

| Mice | Bacteria | Blood | | Brain (100 µl/1000 µl) | |
| --- | --- | --- | --- | --- | --- |
|  |  | volume (ul) | CFU 10^-1^ | weight (g) | CFU 10^0^ |
| WT B6 | K79 | 20 | 33 | 0.44000 | 66 |
| WT B6 | K79 | 20 | 24 | 0.42000 | 77 |
| WT B6 | K79 | 20 | 52 | 0.43000 | 115 |
| WT B6 | K79 | 20 | 18 | 0.44000 | 79 |
| WT B6 | K79 | 20 | 40 | 0.45000 | 83 |
| S1P2 KO | K79 | 20 | 19 | 0.42000 | 10 |
| S1P2 KO | K79 | 20 | 22 | 0.44000 | 12 |
| S1P2 KO | K79 | 20 | 11 | 0.43000 | 22 |
| S1P2 KO | K79 | 20 | 24 | 0.45000 | 14 |
| S1P2 KO | K79 | 20 | 97 | 0.44000 | 6 |

| Mice | Bacteria | Blood (CFU/ml) | Log10 | Brain (CFU/g) |
| --- | --- | --- | --- | --- |
| WT B6 | K79 | 16500 | 4.21748394 | 1500 |
| WT B6 | K79 | 12000 | 4.07918125 | 1833.33333 |
| WT B6 | K79 | 26000 | 4.41497335 | 2674.4186 |
| WT B6 | K79 | 9000 | 3.95424251 | 1795.45455 |
| WT B6 | K79 | 20000 | 4.30103 | 1844.44444 |
| S1P2 KO | K79 | 9500 | 3.97772361 | 238.095238 |
| S1P2 KO | K79 | 11000 | 4.04139269 | 272.727273 |
| S1P2 KO | K79 | 5500 | 3.74036269 | 511.627907 |
| S1P2 KO | K79 | 12000 | 4.07918125 | 311.111111 |
| S1P2 KO | K79 | 48500 | 4.68574174 | 136.363636 |
| p value |  |  | 0.62946446 | 4.6349E-05 |
