# Supplementary material for: Therapeutic development of group B Streptococcus meningitis by targeting a host cell signaling network involving EGFR
Source: EMBO Mol Med. 2021 Jan 21;13(3):e12651. doi: 10.15252/emmm.202012651 (PMC7933950; doi:10.15252/emmm.202012651)
Supplement: Supplementary file 7 — Source Data for Figure 3 [file EMMM-13-e12651-s005.docx]

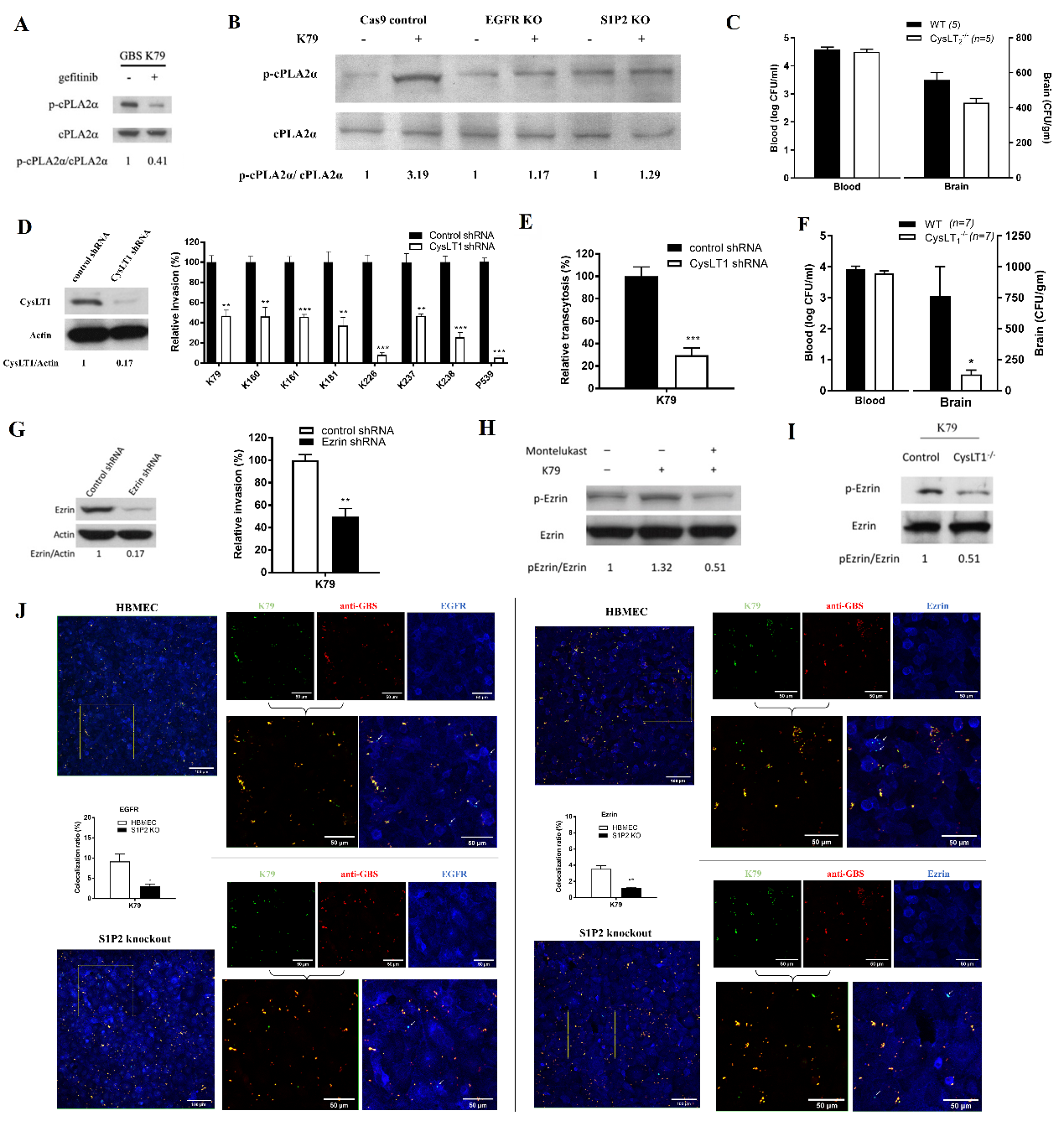


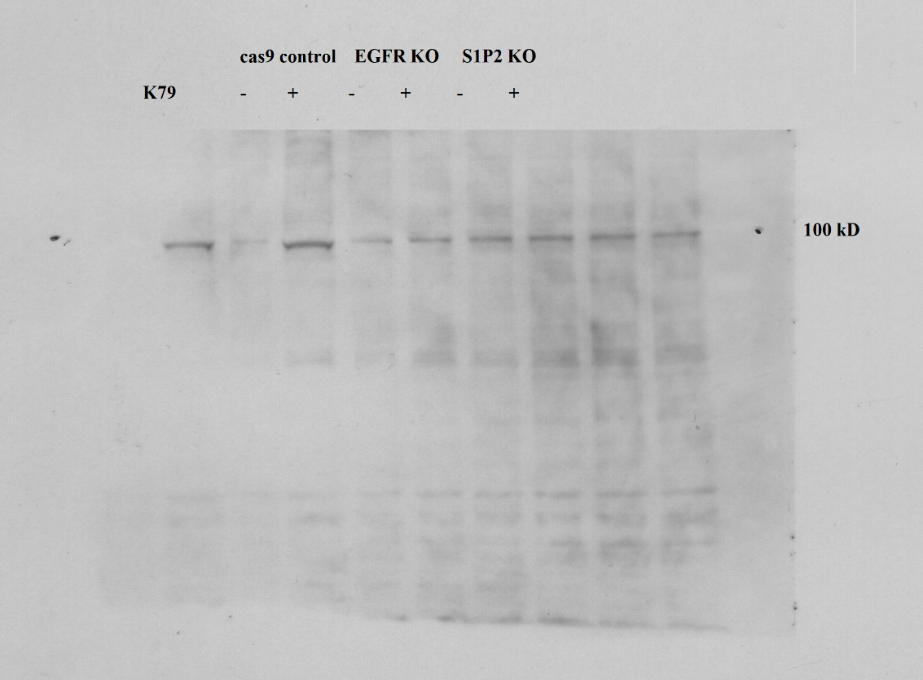
3B Serine phosphorylation of cPLA2α in response to GBS strain K79 in control, EGFR knockout and S1P2 knockout HBMEC

3C Bacterial counts recovered from the blood and brain in wild type mice (n=5) and CysLT2^-/-^ mice (n=5) infected with strain K79 for 1h.

| Mice | Bacteria | Blood | | Brain (100 µl/1000 µl) | |
| --- | --- | --- | --- | --- | --- |
|  |  | volume (ul) | CFU 10^-1^ | weight (g) | CFU 10^0^ |
| WT B6 | K79 | 20 | 90 | 0.42000 | 19 |
| WT B6 | K79 | 20 | 55 | 0.44000 | 30 |
| WT B6 | K79 | 20 | 138 | 0.43000 | 27 |
| WT B6 | K79 | 20 | 84 | 0.45000 | 24 |
| WT B6 | K79 | 20 | 49 | 0.43000 | 18 |
| CysLT2 KO | K79 | 20 | 87 | 0.44000 | 16 |
| CysLT2 KO | K79 | 20 | 38 | 0.45000 | 19 |
| CysLT2 KO | K79 | 20 | 61 | 0.42000 | 20 |
| CysLT2 KO | K79 | 20 | 44 | 0.43000 | 17 |
| CysLT2 KO | K79 | 20 | 122 | 0.43000 | 21 |

| Mice | Bacteria | Blood (CFU/ml) | Log10 | Brain (CFU/g) |
| --- | --- | --- | --- | --- |
| WT B6 | K79 | 45000 | 4.6532125 | 452.38095 |
| WT B6 | K79 | 27500 | 4.4393327 | 681.81818 |
| WT B6 | K79 | 69000 | 4.8388491 | 627.90698 |
| WT B6 | K79 | 42000 | 4.6232493 | 533.33333 |
| WT B6 | K79 | 24500 | 4.3891661 | 418.60465 |
| CysLT2 KO | K79 | 43500 | 4.6384893 | 363.63636 |
| CysLT2 KO | K79 | 19000 | 4.2787536 | 422.22222 |
| CysLT2 KO | K79 | 30500 | 4.4842998 | 476.19048 |
| CysLT2 KO | K79 | 22000 | 4.3424227 | 395.34884 |
| CysLT2 KO | K79 | 61000 | 4.7853298 | 488.37209 |
| p value |  |  | 0.5205892 | 0.0744824 |

3D CysLT1 protein expression in CysLT1 knockdown HBMEC using shRNA (left panel) and relative invasion frequency of 8 GBS isolates in CysLT1 knockdown and control HBMEC.


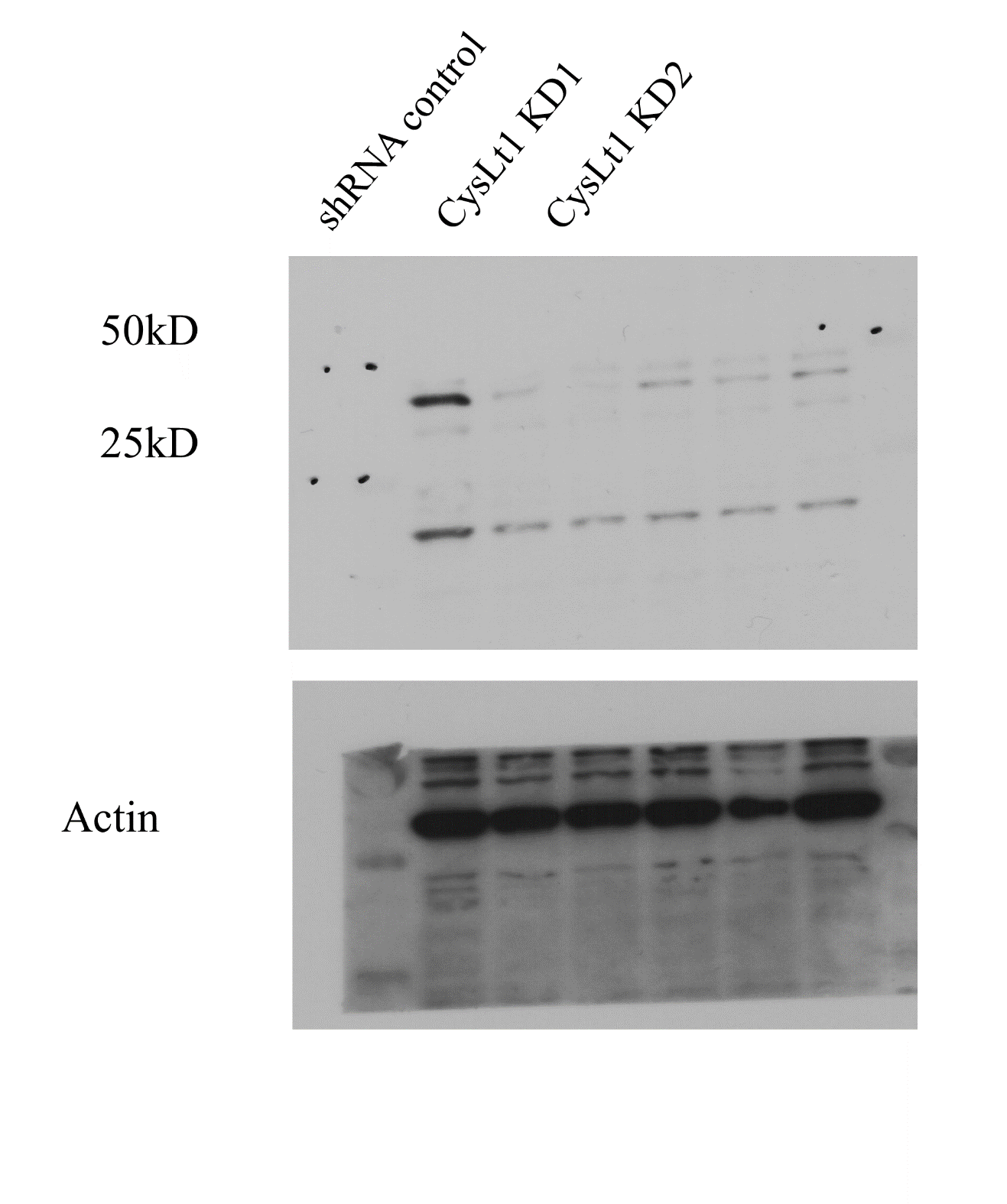


|  | **K79** |  |  |  |  |  |
| --- | --- | --- | --- | --- | --- | --- |
| Genes | dilution | # of colonies | Average | percentage | percentage | pValue |
| GFP | 100 | 178 | 198.3333333 | 90 | 100 | 0.0038689 |
| GFP | 100 | 223 |  | 112 |  |  |
| GFP | 100 | 194 |  | 98 |  |  |
| CysLT1 | 100 | 111 |  | 56 | 47 |  |
| CysLT1 | 100 | 96 |  | 48 |  |  |
| CysLT1 | 100 | 71 |  | 36 |  |  |
|  |  |  |  |  |  |  |
|  | **160** |  |  |  |  |  |
| Genes | dilution | # of colonies | Average | percentage | percentage | pValue |
| GFP | 100 | 67 | 74.66666667 | 90 | 100 | 0.0082121 |
| GFP | 100 | 75 |  | 100 |  |  |
| GFP | 100 | 82 |  | 110 |  |  |
| CysLT1 | 100 | 37 |  | 50 | 46 |  |
| CysLT1 | 100 | 45 |  | 60 |  |  |
| CysLT1 | 100 | 21 |  | 28 |  |  |
|  |  |  |  |  |  |  |
|  | **161** |  |  |  |  |  |
| Genes | dilution | # of colonies | Average | percentage | percentage | pValue |
| GFP | 100 | 175 | 190.3333333 | 92 | 100 | 0.0009143 |
| GFP | 100 | 185 |  | 97 |  |  |
| GFP | 100 | 211 |  | 111 |  |  |
| CysLT1 | 100 | 78 |  | 41 | 46 |  |
| CysLT1 | 100 | 94 |  | 49 |  |  |
| CysLT1 | 100 | 89 |  | 47 |  |  |
|  |  |  |  |  |  |  |
|  | **181** |  |  |  |  |  |
| Genes | dilution | # of colonies | Average | percentage | percentage | pValue |
| GFP | 100 | 141 | 138 | 102 | 100 | 0.0086551 |
| GFP | 100 | 112 |  | 81 |  |  |
| GFP | 100 | 161 |  | 117 |  |  |
| CysLT1 | 100 | 58 |  | 42 | 37 |  |
| CysLT1 | 100 | 29 |  | 21 |  |  |
| CysLT1 | 100 | 66 |  | 48 |  |  |
|  |  |  |  |  |  |  |
|  | **226** |  |  |  |  |  |
| Genes | dilution | # of colonies | Average | percentage | percentage | pValue |
| GFP | 100 | 30 | 34.33333333 | 87 | 100 | 0.0001991 |
| GFP | 100 | 35 |  | 102 |  |  |
| GFP | 100 | 38 |  | 111 |  |  |
| CysLT1 | 100 | 2 |  | 6 | 8 |  |
| CysLT1 | 100 | 2 |  | 6 |  |  |
| CysLT1 | 100 | 4 |  | 12 |  |  |
|  |  |  |  |  |  |  |
|  | **237** |  |  |  |  |  |
| Genes | dilution | # of colonies | Average | percentage | percentage | pValue |
| GFP | 10 | 77 | 76 | 101 | 100 | 0.0039093 |
| GFP | 10 | 64 |  | 84 |  |  |
| GFP | 10 | 87 |  | 114 |  |  |
| CysLT1 | 10 | 34 |  | 45 | 46 |  |
| CysLT1 | 10 | 34 |  | 45 |  |  |
| CysLT1 | 10 | 38 |  | 50 |  |  |
|  |  |  |  |  |  |  |
|  | **238** |  |  |  |  |  |
| Genes | dilution | # of colonies | Average | percentage | percentage | pValue |
| GFP | 100 | 24 | 25 | 96 | 100 | 0.0006571 |
| GFP | 100 | 23 |  | 92 |  |  |
| GFP | 100 | 28 |  | 112 |  |  |
| CysLT1 | 100 | 8 |  | 32 | 25 |  |
| CysLT1 | 100 | 7 |  | 28 |  |  |
| CysLT1 | 100 | 4 |  | 16 |  |  |
|  |  |  |  |  |  |  |
|  | **P539** |  |  |  |  |  |
| Genes | dilution | # of colonies | Average | percentage | percentage | pValue |
| GFP | 100 | 108 | 101.3333333 | 107 | 100 | 1.763E-05 |
| GFP | 100 | 101 |  | 100 |  |  |
| GFP | 100 | 95 |  | 94 |  |  |
| CysLT1 | 100 | 4 |  | 4 | 5 |  |
| CysLT1 | 100 | 4 |  | 4 |  |  |
| CysLT1 | 100 | 8 |  | 8 |  |  |

3E GBS strain K79 traversal of HBMEC monolayer was significantly decreased in CysLT1 knockdown HBMEC compare to control

|  | Dilution | colonies | average | percentage |  | p value |
| --- | --- | --- | --- | --- | --- | --- |
| GFP | 10 | 240 | 217 | 110.59908 | 100 |  |
| GFP | 10 | 191 |  | 88.018433 |  |  |
| GFP | 10 | 220 |  | 101.38249 |  |  |
| CysLt1 KD | 10 | 116 | 104 | 53.456221 | 48 | 0.0020996 |
| CysLt1 KD | 10 | 91 |  | 41.935484 |  |  |
| CysLt1 KD | 10 | 105 |  | 48.387097 |  |  |

3F Bacterial counts recovered from the blood and brain in wild type mice (n=7) and CysLT1^-/-^ mice (n=7) infected with strain K79 for 1h.

| Mice | Bacteria | Blood | | Brain (100 µl/1000 µl) | |
| --- | --- | --- | --- | --- | --- |
|  |  | volume (ul) | CFU 10^-1^ | weight (g) | CFU 10^0^ |
| WT B6 | K79 | 20 | 30 | 0.45 | 64 |
| WT B6 | K79 | 20 | 21 | 0.46 | 14 |
| WT B6 | K79 | 20 | 14 | 0.42 | 78 |
| WT B6 | K79 | 20 | 22 | 0.43 | 27 |
| WT B6 | K79 | 20 | 32 | 0.44 | 22 |
| WT B6 | K79 | 20 | 7 | 0.46 | 10 |
| WT B6 | K79 | 20 | 9 | 0.46 | 20 |
| CysLT1 KO | K79 | 20 | 11 | 0.41 | 3 |
| CysLT1 KO | K79 | 20 | 15 | 0.42 | 5 |
| CysLT1 KO | K79 | 20 | 12 | 0.44 | 13 |
| CysLT1 KO | K79 | 20 | 14 | 0.43 | 9 |
| CysLT1 KO | K79 | 20 | 27 | 0.45 | 7 |
| CysLT1 KO | K79 | 20 | 9 | 0.44 | 2 |
| CysLT1 KO | K79 | 20 | 6 | 0.46 | 2 |

|  |  | Blood (CFU/ml) | Log10 | Brain (CFU/g) |
| --- | --- | --- | --- | --- |
| WT B6 | K79 | 15000 | 4.176091 | 1421.053 |
| WT B6 | K79 | 10600 | 4.025306 | 297.2973 |
| WT B6 | K79 | 6900 | 3.838849 | 1857.143 |
| WT B6 | K79 | 11000 | 4.041393 | 622.2222 |
| WT B6 | K79 | 15750 | 4.197281 | 500 |
| WT B6 | K79 | 3450 | 3.537819 | 225 |
| WT B6 | K79 | 4400 | 3.643453 | 428.5714 |
| CysLT1 KO | K79 | 5600 | 3.748188 | 69.76744 |
| CysLT1 KO | K79 | 7700 | 3.886491 | 111.1111 |
| CysLT1 KO | K79 | 6100 | 3.78533 | 288.8889 |
| CysLT1 KO | K79 | 6900 | 3.838849 | 210.5263 |
| CysLT1 KO | K79 | 13550 | 4.131939 | 146.3415 |
| CysLT1 KO | K79 | 4700 | 3.672098 | 45.45455 |
| CysLT1 KO | K79 | 3200 | 3.50515 | 46.51163 |
| p value |  |  | 0.315819 | 0.020945165 |

3G Relative invasion frequency of GBS strain K79 in ezrin knockdown and control HBMEC

| Genes | dilution | # of colonies | Average | percent | percentage | pValue |
| --- | --- | --- | --- | --- | --- | --- |
| GFP | 100 | 16 | 17.333333 | 92.307692 | 100 |  |
| GFP | 100 | 17 |  | 98.076923 |  |  |
| GFP | 100 | 19 |  | 109.61538 |  |  |
| EZR | 100 | 7 | 8.6666667 | 40.384615 | 50 | 0.0043568 |
| EZR | 100 | 8 |  | 46.153846 |  |  |
| EZR | 100 | 11 |  | 63.461538 |  |  |

3I Ezrin phosphorylation in the homogenates of brain capillaries of CysLT1^-/-^ and control mice infected with GBS strain K79 for 1h


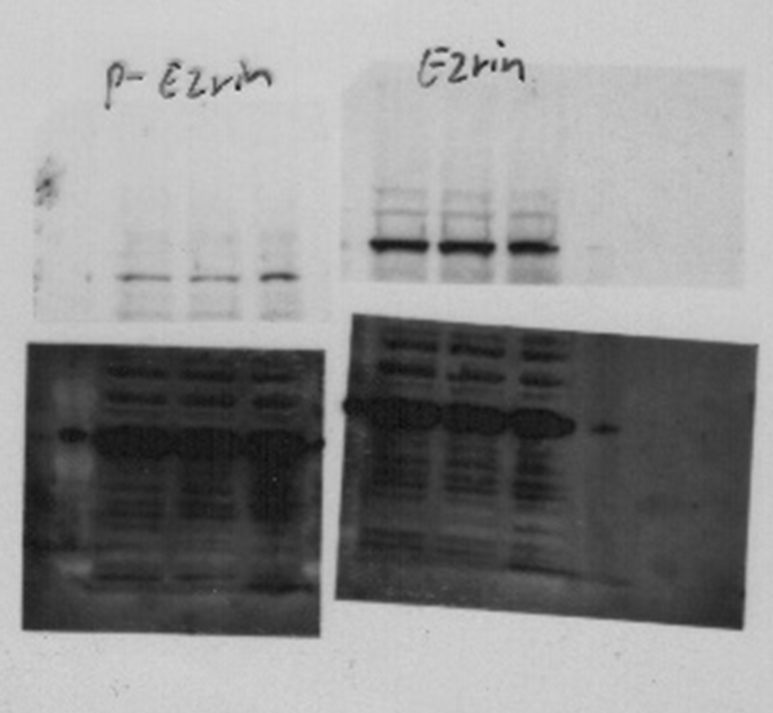


3J Intracellular K79 co-localization with EGFR and ezrin in control and S1P2 knockout HBMEC

| EGFR |  | HBMEC |  |  | S1P2 |  |
| --- | --- | --- | --- | --- | --- | --- |
| Total | 220 | 313 | 295 | 384 | 327 | 358 |
| Colocalized | 15 | 40 | 23 | 8 | 13 | 11 |
| Percentage | 6.8181818 | 12.779553 | 7.796610169 | 2.0833333 | 3.9755352 | 3.0726257 |
| p value | 0.034104 |  |  |  |  |  |
|  |  |  |  |  |  |  |
|  |  |  |  |  |  |  |
| Ezrin |  | HBMEC |  |  | S1P2 |  |
| Total | 373 | 291 | 348 | 267 | 337 | 294 |
| Colocalized | 12 | 9 | 15 | 3 | 5 | 3 |
| Percentage | 3.2171582 | 3.0927835 | 4.310344828 | 1.1235955 | 1.4836795 | 1.0204082 |
| p value | 0.0047895 |  |  |  |  |  |
