# Supplementary material for: Therapeutic development of group B Streptococcus meningitis by targeting a host cell signaling network involving EGFR
Source: EMBO Mol Med. 2021 Jan 21;13(3):e12651. doi: 10.15252/emmm.202012651 (PMC7933950; doi:10.15252/emmm.202012651)
Supplement: Supplementary file 8 — Source Data for Figure 4 [file EMMM-13-e12651-s006.docx]

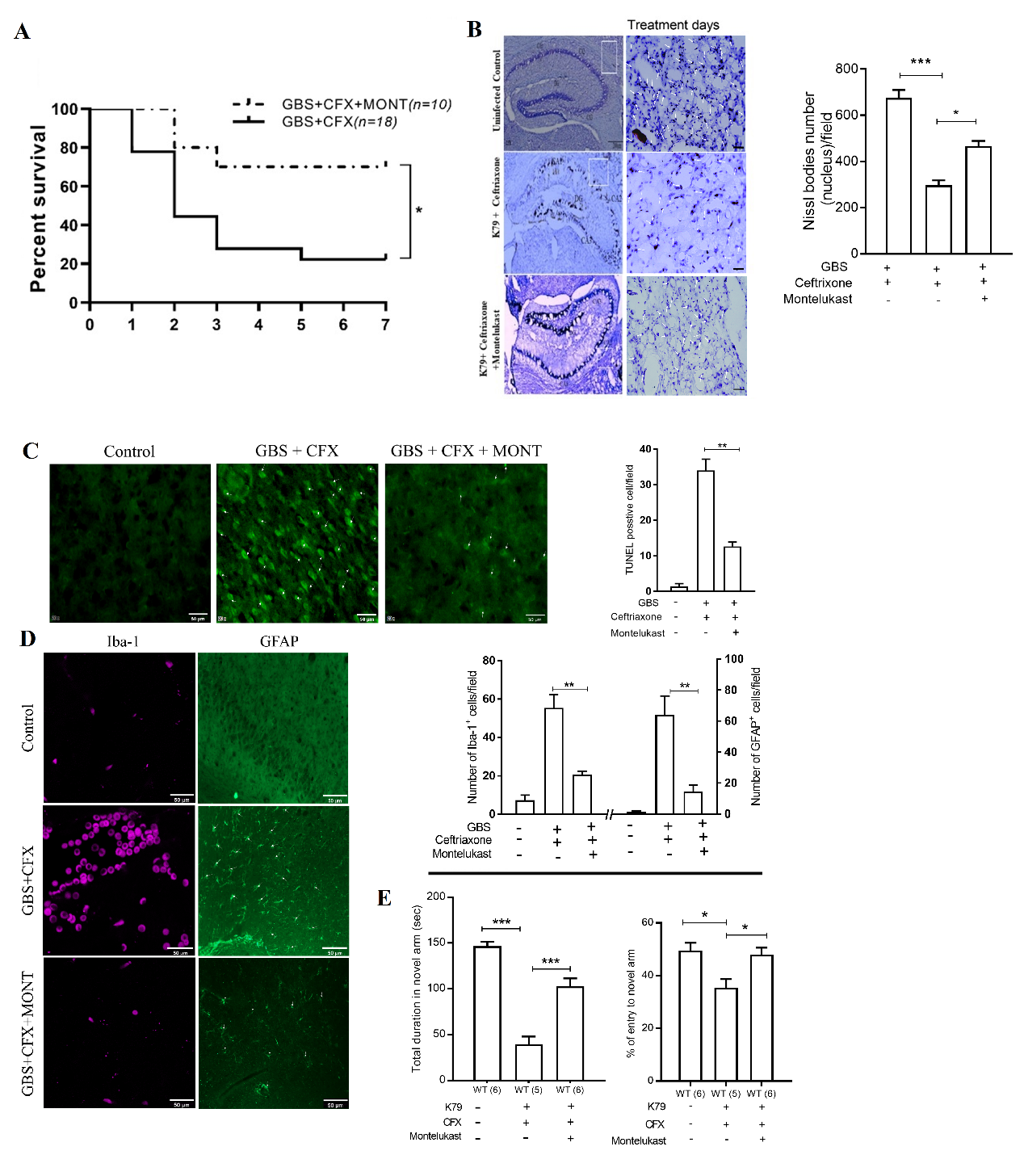


4A Survival of wild type mice receiving drug administration for 7 days after infection with GBS strain K79.

|  | GBS+CFX (n=18) | GBS+CFX+MONT (n=10) |
| --- | --- | --- |
| Day1 | 14 | 8 |
| Day2 | 8 | 7 |
| Day3 | 5 | 7 |
| Day4 | 5 | 7 |
| Day5 | 4 | 7 |
| Day6 | 4 | 7 |
| Day7 | 4 | 7 |
| p value |  | 0.015 |

4B numbers of Nissl-stained bodies

| Nissl stain |  |  |  | p value |
| --- | --- | --- | --- | --- |
| Control | 268 | 285 | 338 |  |
| Ceftriaxone | 435 | 385 | 418 | 0.00021 |
| CFX+MONT | 428 | 467 | 505 | 0.013 |

4C numbers of TUNEL bodies

| TUNEL bodies |  |  |  | p value |
| --- | --- | --- | --- | --- |
| Control | 0 | 1 | 3 |  |
| Ceftriaxone | 28 | 35 | 39 |  |
| CFX+MONT | 15 | 12 | 11 | 0.0012 |

4D numbers of astrocyte and microglia

| GFAP |  |  |  | p value |
| --- | --- | --- | --- | --- |
| Control | 0 | 3 | 1 |  |
| Ceftriaxone | 48 | 56 | 88 |  |
| CFX+MONT | 14 | 8 | 22 | 0.0068 |

| IBA |  |  |  | p value |
| --- | --- | --- | --- | --- |
| Control | 3 | 7 | 12 |  |
| Ceftriaxone | 54 | 68 | 44 |  |
| CFX+MONT | 24 | 20 | 18 | 0.0029 |

4E Y-maze test for spatial learning and memory of different group of wild type mice.

| Total duration in novel arm (sec) |  |  |  |  |  |  | p value |
| --- | --- | --- | --- | --- | --- | --- | --- |
| control | 147 | 150 | 132 | 167 | 142 | 140 |  |
| Ceftriaxone |  | 50 | 34 | 11 | 63 | 38 | 0.00012 |
| CFX+MONT | 124 | 92 | 89 | 102 | 132 | 78 | 0.00017 |
|  |  |  |  |  |  |  |  |
|  |  |  |  |  |  |  |  |
| % of entry to novel arm |  |  |  |  |  |  | p value |
| control | 55.6543 | 46.0453 | 50 | 38.652 | 58.763 | 48.173 |  |
| Ceftriaxone |  | 30.235 | 45.862 | 40.25 | 28.015 | 32.582 | 0.0102 |
| CEF+MONT | 47.198 | 52.65 | 58.34 | 44.765 | 39.654 | 45 | 0.0216 |
